# Two-stage estimation to adjust for treatment switching in randomised trials: a simulation study investigating the use of inverse probability weighting instead of re-censoring

**DOI:** 10.1186/s12874-019-0709-9

**Published:** 2019-03-29

**Authors:** N. R. Latimer, K. R. Abrams, U. Siebert

**Affiliations:** 1 School of Health and Related Research, University of Sheffield, Regent Court, 30 Regent Street, Sheffield, S1 4DA UK; 2 Biostatistics Research Group, Department of Health Sciences, Centre for Medicine, University of Leicester, University Road, Leicester, LE1 7RH UK; 3 UMIT - University for Health Sciences, Medical Informatics and Technology, Eduard-Wallnöfer-Zentrum 1, 6060 Hall in Tirol, Austria; 4 Oncotyrol – Center for Personalized Cancer Medicine, Innsbruck, Austria; 5 Harvard T.H. Chan School of Public Health and Massachusetts General Hospital, Harvard Medical School, Boston, MA USA

**Keywords:** Treatment switching, Treatment crossover, Survival analysis, Overall survival, Oncology, Health technology assessment, Time-to-event outcomes, Prediction, Re-censoring, Inverse probability weighting

## Abstract

**Background:**

Treatment switching is common in randomised trials of oncology treatments, with control group patients switching onto the experimental treatment during follow-up. This distorts an intention-to-treat comparison of the treatments under investigation. Two-stage estimation (TSE) can be used to estimate counterfactual survival times for patients who switch treatments – that is, survival times that would have been observed in the absence of switching. However, when switchers do not die during the study, counterfactual censoring times are estimated, inducing informative censoring. Re-censoring is usually applied alongside TSE to resolve this problem, but results in lost longer-term information – a major concern if the objective is to estimate long-term treatment effects, as is usually the case in health technology assessment. Inverse probability of censoring weights (IPCW) represents an alternative technique for addressing informative censoring but has not before been combined with TSE. We aim to determine whether combining TSE with IPCW (TSEipcw) represents a valid alternative to re-censoring.

**Methods:**

We conducted a simulation study to compare TSEipcw to TSE with and without re-censoring. We simulated 48 scenarios where control group patients could switch onto the experimental treatment, with switching affected by prognosis. We investigated various switching proportions, treatment effects, survival function shapes, disease severities and switcher prognoses. We assessed the alternative TSE applications according to their estimation of control group restricted mean survival (RMST) that would have been observed in the absence of switching up to the end of trial follow-up.

**Results:**

TSEipcw performed well when its weights had a low coefficient of variation, but performed poorly when the coefficient of variation was high. Re-censored analyses usually under-estimated control group RMST, whereas non-re-censored analyses usually produced over-estimates, with bias more serious when the treatment effect was high. In scenarios where TSEipcw performed well, it produced low bias that was often between the two extremes associated with the re-censoring and non-recensoring options.

**Conclusions:**

Treatment switching adjustment analyses using TSE should be conducted with re-censoring, without re-censoring, and with IPCW to explore the sensitivity in results to these application options. This should allow analysts and decision-makers to better interpret the results of adjustment analyses.

**Electronic supplementary material:**

The online version of this article (10.1186/s12874-019-0709-9) contains supplementary material, which is available to authorized users.

## Background

Treatment switching in randomised controlled trials (RCTs) has been shown to be an important issue in health technology assessment (HTA) [1–5]. In oncology trials patients randomised to the control group are often permitted to switch onto the experimental treatment during trial follow-up. This is problematic because it prevents a standard intention-to-treat (ITT) analysis from providing the distinct comparison of randomised treatments that is usually required in HTA. To address this issue, several HTA agencies around the world have embraced statistical adjustment methods [5–8]. These methods allow counterfactual survival times and treatment effects – those that would have been observed had switching not occurred – to be estimated. However, concerns around the use of these methods remain – some agencies are not ready to use adjustment analyses and those that are may still reject an adjustment analysis if it is deemed to be inappropriate [5, 9, 10]. Concerns surround the – often untestable – assumptions made by adjustment methods, but another problem is that each adjustment method can be applied in a multitude of ways [9, 10]. Different applications of the same over-arching method can lead to important differences in results and decision-makers may therefore be concerned about the reliability of analyses presented to them – and, possibly, whether application choices have been made to produce results most favourable to the new treatment. This problem is inhibiting the usefulness of adjustment methods in health care decision making.

**Fig. 1:**
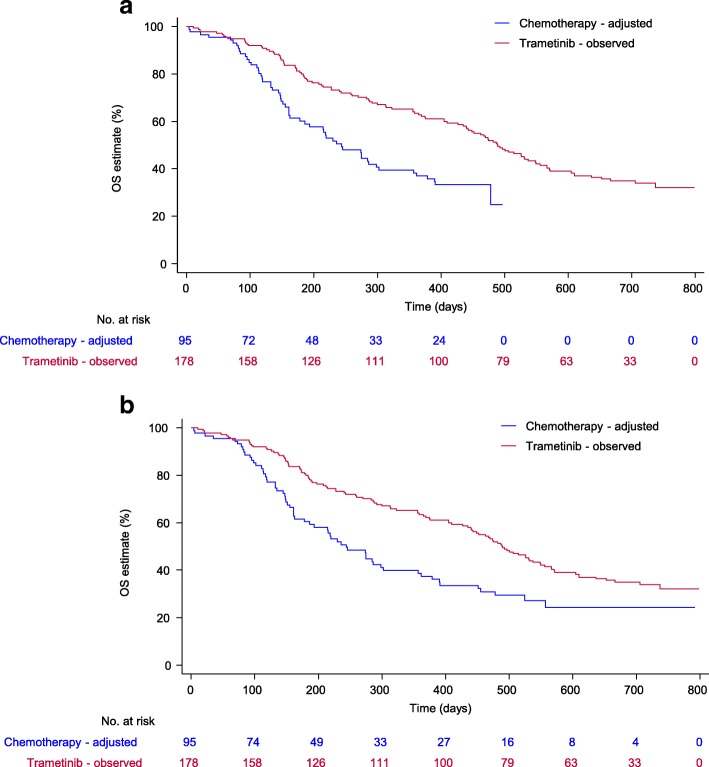
Overall survival in primary efficacy population. **a** Two-stage method with re-censoring; **b** Two-stage method without re-censoring. Adapted from Latimer et al, 2016 [13]

Two-stage estimation (TSE) represents a method for adjusting for treatment switching that has been used in HTA [1, 11, 12]. The method involves estimating counterfactual survival times for patients who switch treatments. Several application choices must be made – such as which accelerated failure time model to use, which covariates to include in that model, and whether or not to include re -censoring. It has been shown that re -censoring – which will be described in the next section – can have a substantial impact on the results of adjustment analyses. Latimer et al. presented a series of adjustment analyses applied to a trial analysing the effect of trametinib compared to chemotherapy in patients with metastatic melanoma, in which 67% of control group patients switched onto the experimental treatment [13]. A standard ITT analysis resulted in a hazard ratio (HR) of 0.72 (95% confidence interval (CI) 0.52 to 0.98), whilst a TSE analysis yielded a HR of 0.43 (95% CI 0.20 to 0.96) when re-censoring was applied, and an HR of 0.53 (95% CI 0.29 to 0.97) without re-censoring – see Fig. 1 for an illustration of the differences between these analyses. Estimates of overall survival often heavily influence estimates of cost-effectiveness and therefore such substantial differences in the point -estimate of the overall survival treatment effect can be crucial in HTA [6, 14–17].

A recently published study investigated the use of re-censoring and concluded that adjustment analyses should be conducted with and without re-censoring [18]. Historically it has been recommended that re-censoring should be applied in adjustment analyses to avoid problems associated with informative censoring in the counterfactual dataset [19–21]. However, re-censoring results in a loss of longer-term information which is problematic when the objective is to estimate long-term survival times and treatment effects – which is almost exclusively the case in HTA [1, 6, 15–17, 21–25]. Latimer et al. found that across a wide range of scenarios analyses that excluded re-censoring consistently produced under-estimates of the longer-term treatment effect, whilst re-censored analyses produced over-estimates of the treatment effect when the survivor function was complex, with decreasing hazards in the longer -term [18]. Whilst this is useful information, it is relevant to question whether alternatives to re -censoring exist, since both re-censoring and non-recensoring options are prone to bias. Inverse probability of censoring weights (IPCW) represents a well-known technique for dealing with informative censoring [26, 27], and has been suggested as an alternative to re-censoring [21], but to our knowledge has never been used specifically for this purpose.

In this paper we investigate the use of IPCW combined with TSE instead of re-censoring, to estimate counterfactual survival times in the presence of treatment switching in an RCT context. We focus on the problem typically seen in HTA [1–5, 7, 9, 10], whereby a subset of control group patients switch onto the experimental treatment after disease progression and we wish to estimate what survival would have been in the control group as a whole had this switching not occurred. Other types of switching occur – switching is sometimes triggered by an interim analysis [28] or patients may switch on to treatments other than those under investigation in the RCT [1, 5]. The type of switching may influence whether adjustment is appropriate, and what the target of estimation should be [29] but these issues are not the focus of this study. We use the simulation study previously reported by Latimer et al. [18] but extend it to include the TSE method in combination with IPCW in addition to the previously investigated TSE with and without re-censoring methods. We aim to establish whether combining TSE with IPCW represents a valid alternative to re-censoring or not re-censoring.

## Methods

The study design has previously been reported by Latimer et al. [18] In this section we first describe the TSE statistical adjustment method and the alternative censoring options, and then summarise the simulation study design.

### Statistical adjustment methods and censoring options

#### Two-stage estimation (TSE)

The TSE adjustment method is designed to adjust for switching that occurs after a specific disease-related time-point (such as disease progression), which is referred to as a “secondary baseline” [11, 12]. Stage one of the TSE method involves estimating the effect of switching on post-secondary baseline survival. Stage two involves using this estimated effect to derive counterfactual survival times for switchers. The method is reliant upon the assumption of no unmeasured confounding – information on prognostic covariates (i.e. independent risk factors for survival which are associated with switching) is required at the secondary baseline time-point [11, 12]. In addition, if switching occurs some time after the secondary baseline, it must be assumed that no time-dependent confounding occurs between the secondary baseline time-point and the time of switch because the TSE only controls for differences between switchers and non-switchers at the secondary baseline time-point [11, 12].

For stage one, post-secondary baseline survival times in control group patients who switch onto the experimental treatment are compared to those in control group patients who do not switch. A parametric accelerated failure time model (e.g. Weibull or Generalised Gamma) is used, controlling for prognostic covariates measured at the secondary baseline time-point and including the switch indicator as a time-dependent variable which equals ‘1’ after the time of switch. This provides an estimate of the treatment effect associated with switching (referred to as *e*^−*ψ*^) in the form of a time-ratio, where *e*^−*ψ*^ represents a multiplicative factor by which an individual’s expected survival time is increased (or decreased) by treatment.

In stage two, the treatment effect estimated in stage one is used in the following counterfactual survival model to estimate counterfactual survival times (*U*_*i*_).

1 $$ {U}_i={T}_{A_i}+{e}^{\psi }{T}_{B_i} $$

The counterfactual survival model splits the observed event time, *T*_*i*_, for each patient into time spent on the control treatment, ${T}_{A_i}$, and time spent on the intervention treatment, ${T}_{B_i}$. For control group switchers, ${T}_{A_i}$ is equal to the time from randomisation until switching occurs, and ${T}_{B_i}$ is equal to the time from switch until death or censoring. To estimate counterfactual survival times for switchers the inverse of the treatment effect estimated in stage 1 (i.e. *e*^*ψ*^) is applied to ${T}_{B_i}$. If switching was estimated to extend survival in stage one, due to effective treatment, this will result in counterfactual survival times being shorter than observed survival times, and vice-versa if switching was estimated to reduce survival in stage one.

#### Re-censoring

Informative censoring is a problem for methods such as TSE that estimate counterfactual survival (and censoring) times [18–21], which is why it is recommended that re-censoring is combined with such methods. For TSE the problem arises because the counterfactual survival model involves adjusting survival times for switchers but not for non-switchers. For some switchers the event time (usually death) may not be observed – instead survival time is censored. For these patients, the TSE involves adjusting censoring times. Thus, the TSE method involves adjusting censoring times in switchers, but not in non-switchers. This will result in informative censoring if there is an association between switching and prognosis.

Re-censoring breaks the dependence between the counterfactual censoring time and switching. Counterfactual survival times associated with a given value of *ψ* (that is, *U*_*i*_(*ψ*)) are re-censored for *all* patients in the control group at the minimum of the administrative censoring time *C*_*i*_ and *C*_*i*_ exp *ψ*, representing the earliest possible censoring time over all possible treatment trajectories, ${D}_i^{\ast}\left(\psi \right)$. *U*_*i*_(*ψ*) is then replaced by ${D}_i^{\ast}\left(\psi \right)$ if ${D}_i^{\ast}\left(\psi \right)<{U}_i\left(\psi \right)$.

#### Alternatives to re-censoring

Evidently, re-censoring results in a loss of longer term survival information – observed events may be re-censored, and follow-up time is lost. This is problematic if the objective is to estimate long-term survival and long-term treatment effects. If the treatment effect changes over time, using re-censored survival data would result in inaccurate estimates of the long-term treatment effect. Similarly, if the objective was to fit parametric survival models to trial data in order to extrapolate into the future (as is often the case in HTA), re-censoring could lead to problems if important changes to the hazard occur beyond the timeframe of the re-censored dataset. Such phenomena are unlikely to be rare – it has often been recognised that cancer populations are characterised by complex hazard functions [30–32]. Fundamentally, whilst re-censoring avoids the problem of informative censoring and produces valid estimates of the treatment effect for the timeframe of the re-censored dataset, it is problematic when the goal is to estimate the long-term survival effect associated with a new treatment.

One alternative to re-censoring is to simply not re-censor. Stages one and two of the TSE method would be applied to obtain counterfactual survival (and censoring) times for switchers and then the analysis would be complete, without the extra step of re-censoring. We denote this method TSEnr. TSEnr is prone to informative censoring in the counterfactual dataset. A second alternative is to use IPCW to address the potentially informative censoring in the counterfactual dataset.

#### TSE combined with inverse probability of censoring weights (TSEipcw)

Combining TSE with IPCW might be beneficial because the loss of information associated with re-censoring is avoided, and the informative censoring associated with not re-censoring is addressed. We denote this TSE and IPCW combination as TSEipcw. Stages one and two of TSE are used to produce a counterfactual dataset for the control group, and then IPCW is applied to adjust for informative censoring. The assumptions associated with TSE are required (switching only after a specified secondary baseline, and no unmeasured confounding). The IPCW part of the approach requires correctly specified weighting models [33], no covariates which ensure (that is, the probability equals 1) that censoring will occur [27, 34, 35], and the ‘no unmeasured confounding’ assumption [34, 36]. After TSE has been used to produce a counterfactual dataset, a model predicting censoring is fit to the group(s) in which switching occurred in order to estimate weights, with all censoring events classified as potentially informative (that is, censoring in switchers *and* non-switchers). IPCW is commonly applied working in discrete time and using pooled logistic regression, meaning that follow-up time is divided up into intervals [27]. The most appropriate time interval duration may depend upon the data being analysed, but could be monthly, weekly, or even daily, and the importance of the chosen duration can be assessed through sensitivity analysis. Stabilised or unstabilised inverse probability of censoring weights can be estimated. Stabilised weights estimated for each individual for each time interval (*t*), as specified by Hernan et al. (2001) are [27]:

2 $$ \widehat{W}(t)={\prod}_{k=0}^t\frac{\mathit{\Pr}\left[C(k)=0|\overline{C}\left(k-1\right)=0,\overline{A}\left(k-1\right),V,T>k\right]}{\mathit{\Pr}\left[C(k)=0|\overline{C}\left(k-1\right)=0,\overline{A}\left(\mathrm{k}-1\right),\overline{L}(k),T>k\right]} $$

where *C*(*k*) is an indicator function demonstrating whether or not censoring had occurred at the end of interval *k*, and $\overline{C}\left(k-1\right)$ denotes censoring history to the end of the previous interval. $\overline{A}\left(k-1\right)$ denotes an individual’s treatment history up to the end of the previous interval, and *V* is an array of an individual’s baseline covariates. $\overline{L}(k)$ denotes the history of an individual’s time-dependent covariates measured at or prior to the beginning of interval *k* and includes *V*. $\overline{L}(k)$ should include baseline and time-dependent covariates that are thought to be prognostic factors for mortality that are also related to switching (and therefore are predictive of censoring time). The numerator of (2) represents the probability of an individual remaining uncensored at the end of interval *k* given that he or she was uncensored at the end of the previous interval (*k* − 1), conditional on baseline characteristics and treatment history. The denominator of (2) represents that same probability, but differs from the numerator because it is conditional on baseline characteristics, treatment history, and time-dependent characteristics. For unstabilised weights, the numerator of (2) is simply replaced with ‘1’.

Once inverse probability of censoring weights have been estimated, they can be used in a weighted survival analysis, accounting for the potentially informative censoring in the counterfactual dataset.

It is relevant to note that in the context being discussed here, treatment history drops out of the weighting model. This may seem strange, given that the rationale for re-censoring is that treatment history predicts counterfactual censoring and survival times – it may be expected that treatment history would be an important covariate in the censoring model. However, we are applying IPCW to a counterfactual dataset in which survival times have been adjusted for treatment switching – survival and censoring times have been estimated as if switching had never occurred. Recall that the censoring model is only fit to the group(s) in which switching occurred – in the context where switching is only from the control group onto the experimental treatment the censoring model is only fit to counterfactual control group data, in which all patients are untreated, leaving no role for treatment history in the model. For the TSEipcw combination to work we need to assume that by including time-updated covariates for factors other than treatment history we can eliminate any informative censoring bias present in the counterfactual dataset.

It is also important to note that IPCW has been used within marginal structural models (MSM) as a standalone approach to adjust for treatment switching, with varying degrees of success, and with the TSE method often producing lower bias than MSM with IPCW [1, 11, 12]. Here we seek to combine TSE and IPCW in order to improve performance in the context of estimating long-term survival.

### Simulation study design

In this study we used the same simulation study design as in our previous study [18], but extended it to investigate whether the TSEipcw method results in an improvement in performance compared to TSE with and without re-censoring. We simulated a subset of the scenarios described in Latimer et al. [18] Datasets with a sample size of 500 were simulated, with 2:1 randomisation in favour of the experimental group, and with treatment switching from the control group onto the experimental treatment permitted. True survival times (without switching) were known. We applied the TSE adjustment methods with and without re-censoring, and TSEipcw, and compared the percentage bias in their estimation of restricted mean survival time (RMST) in the control group, where RMST was the mean survival time restricted to the maximum administrative censoring time in the simulated datasets. We focussed on control group RMST because the objective of the analysis was to estimate survival times for the control group that would have been observed in the absence of treatment switching. We calculated the empirical standard error, root mean squared error and coverage associated with estimates of control group RMST. These measures are defined in the “Performance measures” section below. The simulation study was conducted using Stata software, version 13.1 [37]. The code used for the simulation study is provided in Additional file 1: Appendices A and B.

### Underlying survival times

A joint survival and longitudinal model was used to simultaneously generate a continuous time-dependent covariate (referred to as ‘biomarker’) and survival times [38]. Within the data-generating joint model, the longitudinal model for the underlying biomarker value for the *i*^th^ patient at time *t* was:

3 $$ {biomarker}_i\left(\mathrm{t}\right)={\beta}_{0_i}+{\beta}_1t+{\beta}_2t\times {trt}_i+{\beta}_3{badprog}_i $$

where,

$$ {\beta}_{0_i}\sim N\left({\beta}_0,{\sigma}_0^2\right). $$

Here ${\beta}_{0_i}$ is the random intercept, *β*_1_ is the average rate of change of the biomarker for a patient in the control group, and *β*_1_ + *β*_2_ is the average rate of change of the biomarker for a patient in the experimental treatment group. *trt*_*i*_ is a binary covariate that equals 1 when the patient is in the experimental group and 0 otherwise, *badprog*_*i*_ is a binary covariate that equals 1 when a patient has poor prognosis at baseline and 0 otherwise, and *β*_3_ is the change in the intercept for a patient with a poor prognosis compared to a patient with a good prognosis. Biomarker observations were subject to an error term with a standard normal distribution with mean 0 and variance *σ*, and were simulated to occur at randomisation, and at 21 day intervals thereafter.

We used Crowther and Lambert’s [38] general survival simulation framework to simulate survival dependent on a time-varying biomarker, with a 2-component mixture Weibull baseline survival function, allowing us to simulate complex hazard functions. The model can be written as:

4 $$ {S}_0(t)=p\exp \left(-{\lambda}_1{t}^{\gamma_1}\right)+\left(1-p\right)\exp \left(-{\lambda}_2{t}^{\gamma_2}\right) $$

where *λ*_1_, *λ*_2_ > 0 and *γ*_1_, *γ*_2_ > 0 are scale and shape parameters, respectively. The contribution of the first Weibull to the survival model is represented by *p*, with 0 ≤ *p* ≤ 1, and 1 − *p* represents the contribution of the second Weibull. The related baseline hazard function is:

5 $$ {h}_0(t)=\frac{\lambda_1{\gamma}_1p{t}^{\gamma_1-1}\exp \left(-{\lambda}_1{t}^{\gamma_1}\right)+{\lambda}_2{\gamma}_2\left(1-p\right){t}^{\gamma_2-1}\exp \left(-{\lambda}_2{t}^{\gamma_2}\right)}{p\exp \left(-{\lambda}_1{t}^{\gamma_1}\right)+\left(1-p\right)\exp \left(-{\lambda}_2{t}^{\gamma_2}\right)} $$

The linear predictor of the survival model was incorporated as follows:

6 $$ {h}_i(t)={h}_0(t)\exp \left[{\delta}_1\ {trt}_i+\eta\ \mathrm{t}\times {trt}_i+{\delta}_2\ {badprog}_i+\alpha\ {biomarker}_i(t)\right] $$

with *δ*_1_ representing the direct effect of treatment at time 0, *η* representing the rate at which the direct effect of treatment changes with time, *δ*_2_ representing the impact of poor prognosis, and *α* representing the coefficient of the underlying biomarker level.

Disease progression times were simulated to equal survival times multiplied by a value from a beta distribution with shape parameters (5,10). Disease progression was assumed to be observed at the first simulated consultation following the progression event, with consultations occurring every 21 days.

Random entry into the study was simulated, with the maximum administrative censoring time set at 548 days (1.5 years). Patients in the control group had a random uniform entry time from 0 to 183 days and therefore administrative censoring times ranged from 365 to 548 days.

In our previous study [18], the complexity of the survivor function was an important driver of bias associated with the adjustment methods and therefore we retained a set of scenarios in which the ‘*t*’ terms were excluded from the data generating mechanism, with the resulting survival model being a single – rather than a mixture – Weibull model with a constant treatment effect. The *α* and *η* were set to zero. In these scenarios re-censoring is prone to less bias, because long-term trends in the hazard are established in the short-term and the treatment effect is constant.

In line with our previous study [18], scenarios were ordered such that low numbers were associated with parameter values that were unlikely to result in major biases for the adjustment methods, and high numbers assessed scenarios where bias was more likely to be a problem. For instance, Scenario 1 had a simple, single Weibull survival model, a low treatment effect and a low switching proportion. Scenario 20 provides a more representative illustration of the scenarios tested, characterised by a mixture Weibull survival model and a high, time-dependent treatment effect. Parameter values for the mixture Weibull survival model and the longitudinal biomarker model in Scenario 20 were:

$$ {\beta}_0=20,\kern0.5em {\sigma}_0^2=1, $$ β_1_ = 0.04 , *β*_2_ =  − 0.02, *β*_3_ = 2.5, *σ* = 1, *δ*_1_ =  − 1.30, *δ*_2_ = 0.3, *α* = 0.01, *λ*_1_ = 0.00001, *γ*_1_ = 2.0, *λ*_2_ = 0.00001, *γ*_2_ = 0.8, *p* = 0.5, *η* = 0.003.

**Fig. 2:**
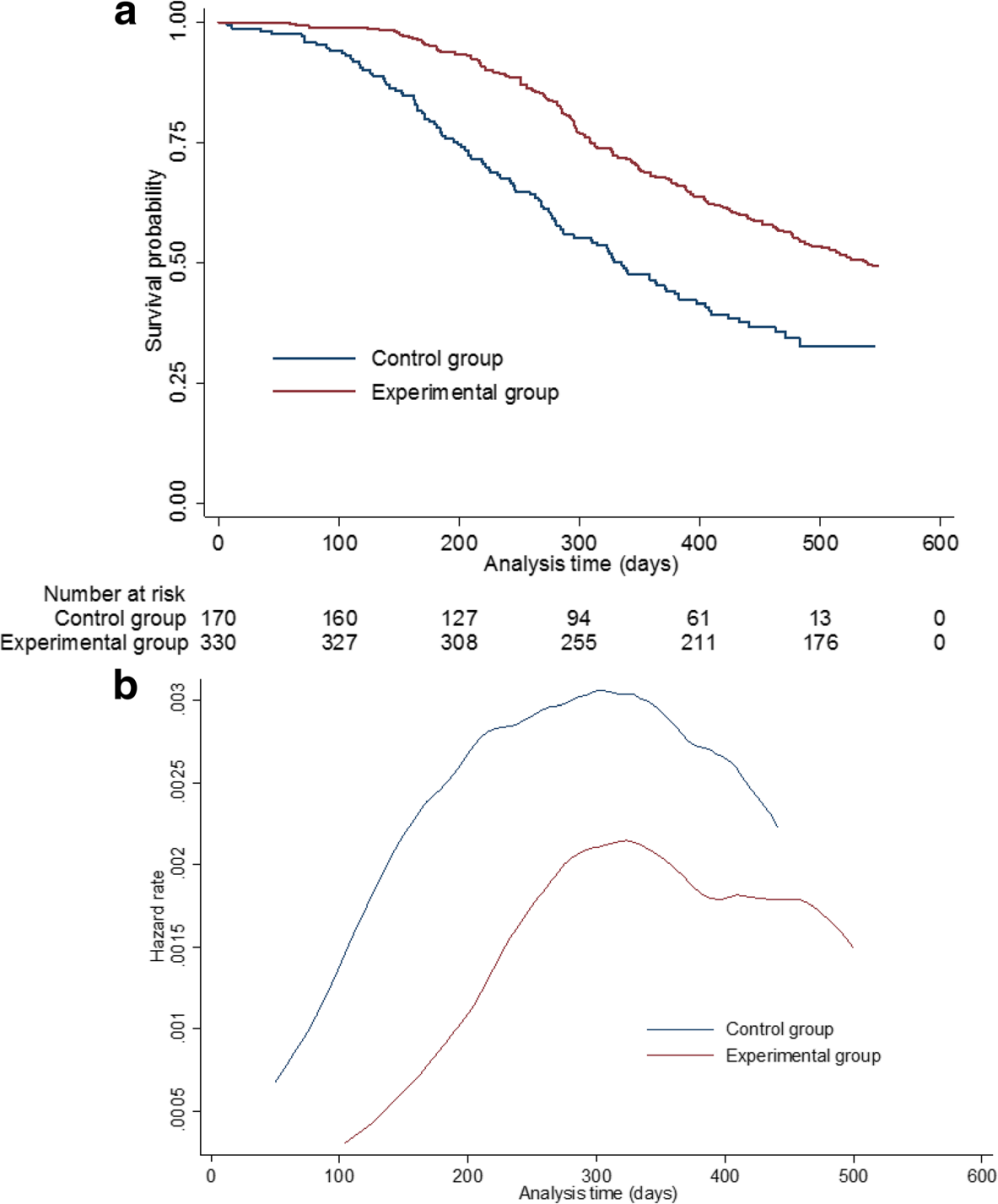
One simulated dataset from Scenario 1 with no switching: (**a**) Overall survival Kaplan–Meier; (**b**) Smoothed hazard rate

Figure 2 presents an example of the Kaplan-Meier curves and hazard function produced by the simulation model (in the absence of treatment switching) from a single simulated data set in Scenario 20. By using a mixture model, we were able to simulate a hazard function that was initially low, then steadily increased before decreasing towards the end of the trial follow-up. As has been previously described, we believe this is reflective of the types of hazards often observed in a metastatic oncology RCT setting [11, 18].

### Treatment effect in the experimental group

For the majority of scenarios, the treatment effect simulated in the experimental group cannot be summarised using a single hazard ratio or acceleration factor, because our hazard function includes ‘*t*’ terms. In reality, we believe that it is likely that the treatment effect (in terms of a hazard ratio) falls over time, as people discontinue treatment, or when only better prognosis patients remain alive. Therefore, primarily we simulated a treatment effect that initially increased during the period of greatest hazard, before falling in the longer-term. In the set of scenarios that excluded the ‘*t*’ terms from the data generating model the true treatment effect was known, with *δ*_1_ representing the log hazard ratio. In other scenarios, as a summary of the size of the treatment effect we calculated the ‘average’ HR and acceleration factors (AF) by generating scenario-specific survival data for a large number of patients (1,000,000) without applying switching, and by fitting Cox and accelerated failure time models to this.

### The switching mechanism

Switching could only occur in control group patients at one of the three consultations immediately following disease progression. A logistic function was used to calculate the probability of switching at these consultations, dependent on the time of observed disease progression and the observed biomarker value at that time-point. Switching probabilities were varied to test different switching proportions, as was the prognosis of patients most likely to switch. Further details on the probability of switching in different simulated groups are presented in Additional file 1: Appendix C.

### Treatment effect in switchers

Switchers were simulated to benefit from switching by multiplying the survival period post switch by a factor (*ω*) using the following approach:

7 $$ {T}_{z_i}={T}_{A_i}+\omega \times {T}_{B_i} $$

where ${T}_{z_i}$ is the survival time incorporating the impact of switching, ${T}_{A_i}$ represents the time of switching and ${T}_{B_i}$ represents the survival time after the switch point that was simulated to occur in the absence of switching. This is the same as the accelerated failure time model presented in (1), but here we denote the treatment effect as *ω* rather than *e*^−*ψ*^.

In our previous study [18], the magnitude of *ω* was varied across scenarios, allowing an assessment of the impact of the ‘common treatment effect’ assumption, whereby the effect of the treatment is assumed to be the same irrespective of when it is received. This was important because one of the methods tested in our previous study, the rank preserving structural failure time model (RPSFTM), assumes that there is a common treatment effect. In contrast, the TSE method (and IPCW) has been shown to be unaffected by this assumption [11, 12, 18], and therefore in the present study we did not vary *ω*. Instead we set *ω* equal to the average acceleration factor observed in the experimental group. Scenario-specific survival data were generated for 1,000,000 patients without applying switching and an accelerated failure time model was applied to estimate *ψ*, with *ω* then set to equal *e*^−*ψ*^.

### Scenarios investigated

Our intention was to investigate realistic scenarios and to test the sensitivities of the adjustment methods to changes in key scenario characteristics. Scenarios were run varying the following characteristics:

- Switch proportion: low (approximately 25% of control group patients who experienced disease progression); moderate (approximately 55% of control group patients who experienced disease progression)
- Treatment effect: low (average HR/AF/ *ψ* under the incorrect assumption of proportional treatment effects approximately 0.80/1.13/− 0.12); high (average HR/AF/ *ψ* approximately 0.56/1.85/− 0.62)
- Switcher prognosis: good prognosis more likely to switch; poor prognosis more likely to switch;
- Severity of disease: low (restricted mean survival in control group approximately 357 days, administrative censoring proportion approximately 40–50%); high (restricted mean survival in control group approximately 228 days, administrative censoring proportion approximately 17–25%)
- Complexity of the survivor function and time dependency of treatment effect: simple (single Weibull model, α = 0.00, η = 0.000); moderate (mixture Weibull model, α = 0.01, η = 0.003); high (mixture Weibull model, α = 0.01, η = 0.006)

Using a 2x2x2x2x3 factorial design resulted in a total of 48 scenarios. The scenarios were numbered 1–48 with all levels of one factor nested inside one level of the next factor, following the order listed above. This represents half of the scenarios reported in Latimer et al. [18] because in this study we did not include scenarios to test the sensitivity of the results to the common treatment effect assumption, because none of the included methods rely upon this assumption. It is important to note that scenarios are ordered differently in the present study, to aid the presentation of results. For instance, Scenarios 1, 2, 3 and 4 in this study are equivalent to Scenarios 3, 51, 7 and 55 in Latimer et al. [18] One thousand simulations were run for each scenario. Further details on scenario values and settings are presented in Additional file 1: Appendices D and E.

### Adjustment methods compared

We applied TSE using a Weibull model with disease progression as the secondary baseline time-point, and included covariates for switching, baseline prognosis group, observed biomarker value at time 0, observed time-to-disease progression, and observed biomarker value at disease progression. We included the two-stage method with and without re-censoring (denoted as TSE and TSEnr respectively). For TSEipcw, we applied TSEnr to estimate counterfactual survival and censoring times for switchers, and then estimated weights according to eq. (2) using a similar approach to that described by Fewell et al. [39] Covariates included in *V* were baseline prognosis group and the biomarker value observed at time 0. Additional covariates included in $\overline{L}(k)$ were an indicator for disease progression, observed time-to-disease progression, and observed biomarker value at the beginning of the interval. 21-day intervals were used, representing the observation times simulated in our data, and time was incorporated using restricted cubic splines using the Stata command rcsgen. Interior knots were placed at the 33rd and 67th centiles of the distribution of censoring times and 2 boundary knots were placed at the minimum and maximum values of the censoring times.

For IPCW applications that use stabilised weights, baseline covariates should be incorporated in survival models fitted to the weighted survival times. This is computationally intensive in a simulation setting when estimating mean survival times. Therefore, in the majority of scenarios we applied TSEipcw using only unstabilised weights. However, in Scenarios 17–20 and 25–28 we applied TSEipcw with stabilised and unstabilised weights. This allowed us to investigate the impact of stabilised compared to unstabilised weights in scenarios deemed realistic (that is, with a moderately complex survivor function).

To provide context on the performance of the various TSE applications, we included a ‘No Switching’ analysis, representing the results of a standard ITT analysis (that is, an unadjusted estimate of control group RMST) undertaken on the simulated dataset before switching was applied. This does not represent a feasible estimator, but provides a useful upper bound for adjustment method performance which may be considered a ‘gold standard’. We also included a standard ITT analysis after switching has been applied.

### Performance measures

Control group restricted mean survival time (RMST) was our estimand upon which we based our performance measures. This is in line with our aim of investigating the performance of adjustment methods in estimating counterfactual survival times in the presence of switching from the control group onto the experimental treatment. As previously described by Latimer et al. [18] our simulated survival function was not analytically tractable so for each scenario we simulated data for 1,000,000 patients without incorporating treatment switching, and estimated the RMST at 548 days (the maximum administrative censoring time in the simulated datasets). This value is the product of a simulation rather than a calculation so is prone to error, but this is minimal given the large number of patients simulated. For instance, in Scenario 1 the standard error of the control group “true” RMST was 0.28 days (0.07% of the estimated RMST).

To estimate RMST at 548 days for each of the adjustment methods we used flexible parametric models [40]. Non-parametric methods could not be used because when re-censoring is applied longer-term information is lost and therefore a non-parametric estimate of RMST would be restricted to too short a time period. Instead, we used flexible parametric models fitted to the counterfactual datasets provided by TSE and TSEnr, and fitted to the weighted survival times provided by TSEipcw. These were used to obtain the survivor function extrapolated to 548 days, ensuring our RMST comparisons were comparing “like with like”. The Stata command stpm2 was used to fit the models on the log cumulative hazard scale, with 3 interior knots placed at the 25th, 50th and 75th centiles of the distribution of log survival times and boundary knots placed at the minimum and maximum of the distribution of uncensored survival times [40]. If the final observed counterfactual survival time was less than 548 days, RMST at 548 days was estimated through extrapolation using stpm2, which involves a linear extrapolation of log time on the log cumulative hazard scale, based on the fitted function and extrapolating from the last knot. This is consistent with UK HTA recommendations for undertaking survival modelling in the presence of complex hazard functions [41, 42].

To appropriately estimate confidence intervals for RMST for TSE and other adjustment methods the entire adjustment process should be bootstrapped, in order to take into account the uncertainty in underlying survival times as well as the uncertainty associated with the adjustment. This involves sampling from the dataset being analysed multiple times, applying the adjustment method and estimating RMST for each sample. In a simulation study setting, with 1000 simulated datasets generated for each scenario, this is extremely computationally intensive. We completed this procedure only for the four key scenarios that we focus on in the Results section. For each simulation we took 200 samples, sampled with replacement, clustered by individual patient, stratified by treatment group and using the same sample size as the simulated datasets (i.e. 500). The adjustment method was applied to each sample and RMST was calculated. Confidence intervals were then estimated using the 2.5th and 97.5th percentiles of the calculated RMST over the 200 bootstrap samples for each simulation.

The performance of methods was evaluated according to the percentage bias in their estimate of control group RMST at 548 days. Percentage bias was estimated by taking the difference between the mean estimated RMST and the true RMST and expressing this as a percentage of true RMST [43]. The root mean squared error (RMSE) of the percentage bias was calculated to provide information on the variability of estimates in combination with percentage bias. The empirical standard error (SE) of the RMST estimate was also calculated for each method, defined as the standard deviation in the percentage bias of the RMST estimate for each method over the 1000 simulations run for each scenario. Coverage was also calculated, defined as the proportion of simulations where the 95% confidence interval of the RMST contained the true RMST. Convergence was measured, defined as the proportion of times that each method resulted in an estimate of control group RMST. Percentage bias, RMSE, empirical SE and coverage were calculated based upon simulations in which convergence occurred. For each method, Monte Carlo (MC) standard errors were calculated for each performance measure, in line with Morris et al. [44] For methods that incorporated IPCW, we recorded the proportion of simulations in each Scenario that resulted in maximum weights that were greater than (i) 100 and (ii) 1000. We also recorded the coefficient of variation of the weights (that is, the standard deviation of the weights divided by the mean of the weights) measured across control group patients in each simulated data set. This allowed us to explore the relationship between variations in the weights and the performance of the TSEipcw method.

## Results

Results for TSE and TSEnr have been previously reported [18] – to summarise, TSEnr produced positive bias across almost all scenarios, over-estimating control group RMST and therefore under-estimating the true treatment effect. TSE always produced lower estimates of RMST than TSEnr, consistently producing negative bias and over-estimating the true treatment effect. Neither method consistently outperformed the other with respect to bias in RMST estimates, but TSEnr produced lower RMSE in every scenario, demonstrating greater precision than TSE. Here, we focus on the relative performance of the TSEipcw method. We present detailed results from four key scenarios that illustrate the key findings. We then summarise the extent to which these reflect the results of the other scenarios simulated. Finally we report findings comparing TSEipcw with stabilised and unstabilised weights, and TSEipcw results according to the coefficient of variation in the weights.

A summary table describing the characteristics of each scenario is presented in Additional file 1: Appendix F. Additional file 1: Appendices G, H and I present the percentage bias, empirical standard error and RMSE respectively across all scenarios for each method.

### Detailed results from key scenarios

Table 1 presents detailed results from Scenarios 20 and 28. The characteristics of Scenario 20, with regard to survival times, switch proportion, treatment effect and censoring proportion are described in Table 1. This scenario incorporated a large treatment effect, a moderate switch proportion, and relatively low disease severity (and therefore a high censoring proportion). As expected, the ITT analysis over-estimated control group RMST, equivalent to a percentage bias of 7.5%. The TSE analysis that incorporated re-censoring under-estimated control group RMST (percentage bias − 2.3%) and TSEnr over-estimated control group RMST (percentage bias 3.5%). TSEipcw led to a higher level of percentage bias than the other adjustment methods (percentage bias − 5.1%). The mean coefficient of variation of the inverse probability of censoring weights was 16.9, and the maximum weight was greater than 100 in 77.4% of simulations (and greater than 1000 in 43.5% of simulations).

**Table 1:** Scenarios 20 and 28 – performance measures for estimation of control arm RMST

Scenario details	Method	Percent bias	Empirical SE of % bias	RMSE of % bias	Coverage (%)	Convergence (%)
Scenario number: 20 True RMST: Control: 357 Experimental: 430 Mean switch: 57% True ave. HR: 0.57 True ave. AF: 1.53 Mean censored: 50%	No switching	0.0	3.7	3.7	94.4	100.0
	ITT	7.5	3.4	8.3	46.7	100.0
	TSE	−2.3	6.9	7.3	97.8	100.0
	TSEnr	3.5	3.9	5.3	86.1	100.0
	TSEipcw	−5.1	16.3	17.1	96.0	95.8
	min/max MC error	0.1/0.5	0.1/0.4	0.1/0.5	0.5/1.6	–
Scenario number: 28 True RMST: Control: 228 Experimental: 322 Mean switch: 57% True ave. HR: 0.56 True ave. AF: 1.85 Mean censored: 26%	No switching	−0.1	5.7	5.7	94.7	100.0
	ITT	15.1	5.5	16.0	29.1	100.0
	TSE	−3.5	9.1	9.8	93.0	100.0
	TSEnr	4.0	6.5	7.6	91.6	100.0
	TSEipcw	1.1	11.3	11.3	97.1	99.9
	min/max MC error	0.2/0.4	0.1/0.3	0.1/0.8	0.5/1.4	–

Note: *RMST* restricted mean survival time, *HR* hazard ratio, *AF* acceleration factor, *SE* standard error, *RMSE* root mean squared error, *MC* Monte-Carlo, *ITT* intention to treat, *TSE* two-stage estimation, *TSEnr* two-stage estimation without re-censoring, *TSEipcw* two-stage estimation with inverse probability of censoring weights

The only substantive difference between Scenario 20 and Scenario 28 was that disease severity was greater in Scenario 28, leading to the censoring proportion being approximately halved. The TSE and TSEnr methods were relatively unaffected by this change (percentage bias − 3.5 and 4.0% respectively), but the percentage bias produced by TSEipcw reduced substantially (percentage bias 1.1%), such that it was lower than that produced by TSE and TSEnr. Whilst TSE and TSEnr under- and over-estimated control group RMST respectively, TSEipcw produced an RMST estimate that was between those two extremes. In Scenario 28 the mean coefficient of variation of the inverse probability of censoring weights was 5.4, substantially lower than that observed in Scenario 20. The maximum weight was greater than 100 in 53.5% of simulations and was greater than 1000 in 10.6% of simulations.

Table 2 presents detailed results from Scenarios 25 and 26. Scenario 26 was approximately equivalent to Scenario 28, except the treatment effect was lower. Scenario 25 was approximately equivalent to Scenario 28 except the treatment effect was lower and the switching proportion was reduced to approximately 25% of at-risk patients. TSEipcw performed well in both of these scenarios, producing percentage bias of 1.8% in Scenario 26 and 1.0% in Scenario 25. The mean coefficient of variation of the inverse probability of censoring weights was 1.5 in Scenario 26 and 0.9 in Scenario 25. The maximum weight was greater than 100 in 4.7% of simulations in Scenario 25, but was never greater than 1000. In Scenario 26 the maximum weight was greater than 100 in 21.2% of simulations and greater than 1000 in 0.3%. Although TSEipcw produced low bias in Scenarios 25 and 26, TSE and TSEnr produced similar or lower bias (percentage bias 0.3% in both scenarios for TSE, and 1.5 and 1.0% for Scenarios 26 and 25 respectively for TSEnr).

**Table 2:** Scenarios 25 and 26 – performance measures for estimation of control arm RMST

Scenario details	Method	Percent bias	Empirical SE of % bias	RMSE of % bias	Coverage (%)	Convergence (%)
Scenario number: 25 True RMST: Control: 228 Experimental: 269 Mean switch: 25% True ave. HR: 0.78 True ave. AF: 1.30 Mean censored: 18%	No switching	0.1	5.8	5.8	94.9	100
	ITT	2.7	5.8	6.4	92.9	100
	TSE	0.3	6.4	6.4	95.6	100
	TSEnr	1.0	5.9	6.0	94.8	100
	TSEipcw	1.0	6.6	6.7	95.3	100
	min/max MC error	0.2/0.2	0.1/0.1	0.1/0.2	0.7/0.8	–
Scenario number: 26 True RMST: Control: 228 Experimental: 269 Mean switch: 57% True ave. HR: 0.78 True ave. AF: 1.30 Mean censored: 18%	No switching	0.0	5.7	5.7	95.6	100
	ITT	6.2	5.6	8.4	85.4	100
	TSE	0.3	7.1	7.1	95.2	100
	TSEnr	1.5	6.4	6.6	95.3	100
	TSEipcw	1.8	8.3	8.5	95.2	100
	min/max MC error	0.2/0.3	0.1/0.2	0.1/0.3	0.6/1.2	–

Note: *RMST* restricted mean survival time, *HR* hazard ratio, *AF* acceleration factor, *SE* standard error, *RMSE* root mean squared error, *MC* Monte-Carlo; *ITT* intention to treat, *TSE* two-stage estimation, *TSEnr* two-stage estimation without re-censoring, *TSEipcw* two-stage estimation with inverse probability of censoring weights

The RMSE results presented in Tables 1 and 2 demonstrate that the levels of variability associated with the different adjustment methods differed importantly – as also demonstrated in our previous study [18]. TSEnr produced least RMSE (aside from the gold standard ‘no switching’ analysis) in all four scenarios but did not produce least bias in any of the scenarios. TSEipcw produced slightly higher levels of RMSE than TSE in the three scenarios in which the mean coefficient of variation of the inverse probability of censoring weights was relatively low (Scenarios 25, 26 and 28), but produced substantially higher RMSE in Scenario 20, in which the mean coefficient of variation of the weights was high. The RMSE results reflect the fact that the empirical standard errors of the percentage bias differed substantially between methods. As shown in our previous study [18], TSE produced empirical standard errors that were substantially higher than those associated with TSEnr. These were higher still for TSEipcw in the four scenarios considered here – with their size again seemingly related to the size of the mean coefficient of variation of the inverse probability of censoring weights.

Tables 1 and 2 show that coverage was around 95–96% for all the adjustment methods in Scenarios 25 and 26, in which percentage bias was low for all methods. Coverage changed in Scenarios 20 and 28, in which levels of percentage bias were generally slightly higher than in Scenarios 25 and 26. In Scenario 28 coverage for TSE and TSEnr decreased to 92–93%. In Scenario 20, which differed to Scenario 28 by having a higher censoring proportion, coverage associated with TSEnr decreased to 86.1%, whilst remaining over 95% for TSE and TSEipcw, despite percentage biases of − 2.3% and − 5.1% respectively. This indicates that for these methods model standard errors must overestimate the empirical standard error, because coverage is adequate despite bias. Coverage was markedly better for the adjustment methods than for the ITT analysis. This is in contrast to results from previous simulation studies where we did not use bootstrapping to estimate confidence intervals for the adjustment methods [11, 12, 18]. Convergence was achieved with all of the adjustment methods, with the possible exception of the TSEipcw. Whilst the method converged successfully, in doing so it resulted in weights with a large range in some simulations, particularly in Scenario 20.

### Results from other scenarios

**Fig. 3:**
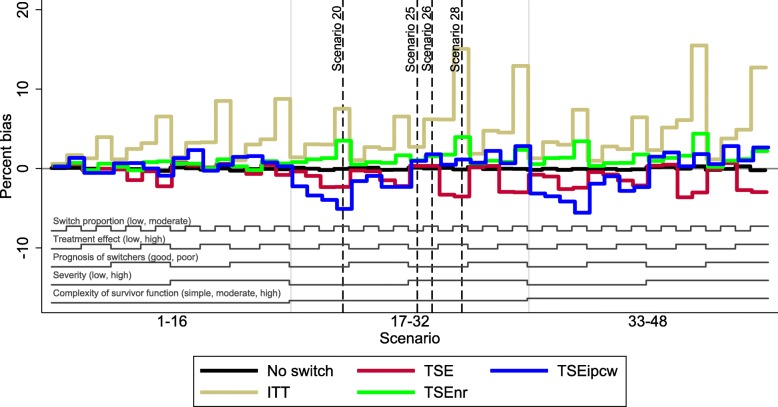
Percentage bias across all scenarios. Note: ITT: intention to treat; TSE: two-stage estimation; TSEnr: two-stage estimation without re-censoring; TSEipcw: two-stage estimation with inverse probability of censoring weights

**Fig. 4:**
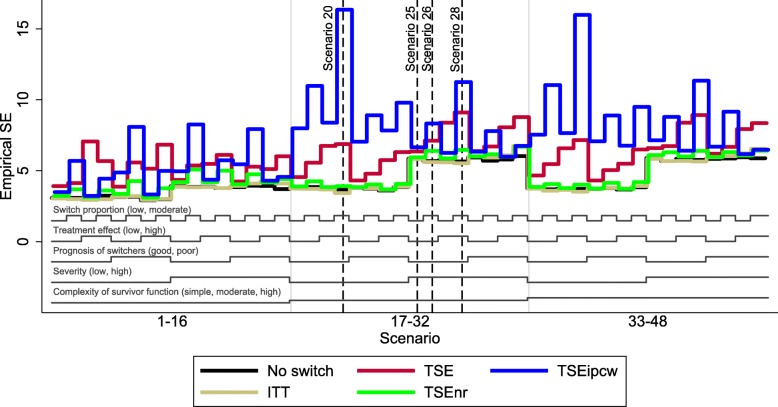
Empirical standard error across all scenarios. Note: ITT: intention to treat; TSE: two-stage estimation; TSEnr: two-stage estimation without re-censoring; TSEipcw: two-stage estimation with inverse probability of censoring weights. SE: standard error

**Fig. 5:**
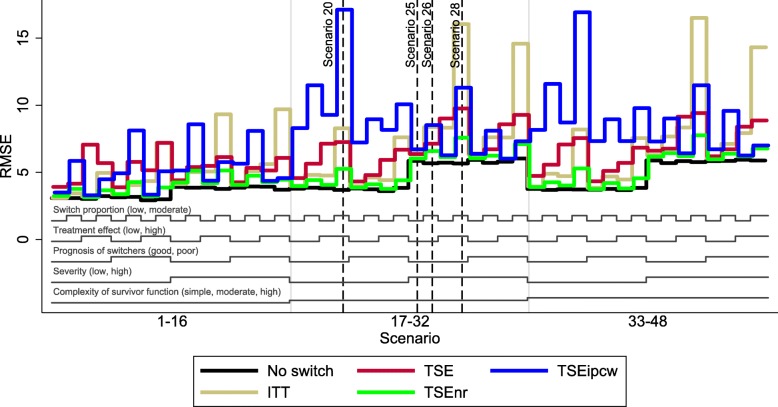
Root mean squared error across all scenarios. Note: ITT: intention to treat; TSE: two-stage estimation; TSEnr: two-stage estimation without re-censoring; TSEipcw: two-stage estimation with inverse probability of censoring weights. RMSE: root mean squared error

The overall patterns in our results are illustrated in Figs. 3, 4, and 5, which present nested loop plots for percentage bias, empirical SE and RMSE [45]. More detailed barplots for each of these performance measures are presented in Additional file 1: Appendices G, H and I. The results presented for Scenarios 20, 25, 26 and 28 provide a good basis for reporting the results of the remaining scenarios – particularly those observed in scenarios where the complexity of the survivor function was moderate or high. The characteristics that had the most impact on the performance of TSEipcw were the complexity of the survivor function and the severity of disease. The complexity of the survivor function, the switching proportion and the size of the treatment effect were particularly important for TSE and TSEnr. The prognosis of switchers was not an important driver of the results.

TSE, TSEnr and TSEipcw all produced low levels of bias in Scenarios 1–16, in which the survivor function was simple, with a constant treatment effect over time. TSE produced least percentage bias in 6 of these scenarios, and TSEnr and TSEipcw produced least bias in 5 apiece. TSEnr consistently produced lower empirical SE and RMSE than TSEnr and TSEipcw, and TSEipcw produced lower empirical SE and RMSE than TSE in 9 of the 16 scenarios. The mean coefficient of variation in the inverse probability of censoring weights ranged between 0.6 and 2.3 in these scenarios, the proportion of simulations in which the maximum weight was greater than 100 ranged between 1.0 and 34.2% and the proportion of simulations in which the maximum weight was greater than 1000 ranged between 0 and 1.5%.

When the complexity of the survivor function was moderate or high, with a decreasing treatment effect over time, the performance of the adjustment methods varied much more widely. Scenario 20 provides a useful representation of the scenarios in which disease severity was low (that is, Scenarios 17–24 and Scenarios 33–40), and Scenarios 25, 26 and 28 reflect the findings in scenarios in which disease severity was high (Scenarios 25–32 and 41–48). Disease severity did not have an important effect on the performance of the TSE and TSEnr methods, but had a large impact on the TSEipcw method. When disease severity was low, resulting in a relatively high degree of censoring (approximately 40–50%), TSEipcw consistently produced more bias and considerably higher empirical SE and RMSE than TSE and TSEnr. In these scenarios, the mean coefficient of variation in the inverse probability of censoring weights ranged between 4.7 and 16.9, the proportion of simulations in which the maximum weight was greater than 100 ranged between 37.7 and 77.4%, and the proportion of simulations in which the maximum weight was greater than 1000 ranged between 8.1 and 44.1%. In contrast, when disease severity was high, resulting in relatively low censoring proportions (approximately 17–25%), TSEipcw often produced similar or lower levels of bias than TSE and TSEnr. In these scenarios, TSE produced least bias in 8 scenarios, TSEnr produced least bias in 2 scenarios and TSEipcw produced least bias in 6 scenarios. TSEnr continued to consistently produce the lowest empirical SE and RMSE. TSEipcw produced lower empirical SE and RMSE than TSE in approximately half of these scenarios. The mean coefficient of variation in the inverse probability of censoring weights ranged between 0.7 and 5.5 in these scenarios, whilst the proportion of simulations in which the maximum weight was greater than 100 ranged between 1.5 and 53.5%, and the proportion of simulations in which the maximum weight was greater than 1000 ranged between 0.0 and 11.6%.

Whilst TSEipcw consistently produced low levels of bias in scenarios in which disease severity was high (resulting in low coefficients of variation in the inverse probability of censoring weights, and few simulations with very high maximum weights), it did not consistently do better than TSE and TSEnr in these scenarios – often similar levels of bias were produced by each of the methods. As shown in our previous study [18], TSE and TSEnr are prone to higher levels of bias when the treatment effect and/or the switching proportion is high. Generally, TSEipcw produced lower percentage bias than TSE and TSEnr when there was a moderate or complex survivor function and a high treatment effect, provided disease severity was high, as demonstrated by Scenario 28. When the treatment effect was low, TSE consistently produced least bias, with TSEnr and TSEipcw producing similar, marginally higher levels of bias – as demonstrated by Scenarios 25 and 26.

### Stabilised vs Unstabilised weights

Percentage bias, empirical standard error and RMSE results for applications of TSEipcw with and without stabilised weights across Scenarios 17–20 and 25–28 are presented in Additional file 1: Appendix J. Applications of TSEipcw that used stabilised weights generally produced marginally lower percentage bias than those that used unstabilised weights in scenarios with a low disease severity – that is, in the scenarios where TSEipcw performed poorly with unstabilised weights. However, whilst RMSE and mean coefficients of variation in the weights were substantially reduced in these scenarios (with mean coefficients of variation ranging from 5.6 to 16.8 for applications with unstabilised weights and 1.8 to 12.0 for stabilised weights), percentage bias and RMSE remained relatively high compared to TSE and TSEnr. The proportion of simulations with maximum weights over 100 was reduced in these scenarios when stabilised weights were used, but remained high (ranging from 44.6 to 77.4% with unstabilised weights and from 15.1 to 60.9% with stabilised weights).

In scenarios with a high disease severity, TSEipcw analyses that incorporated stabilised weights generally produced similar levels of percentage bias compared to applications that incorporated unstabilised weights, and RMSE were relatively unaffected. Mean coefficients of variation were reduced (ranging from 0.9 to 5.4 for applications with unstabilised weights, and from 0.1 to 0.9 for stabilised weights), as was the proportion of simulations with maximum weights over 100 (ranging from 4.7 to 53.5% with unstabilised weights and from 0.0 to 7.3% with stabilised weights). This did not translate to appreciably improved performance except in Scenario 28, which was the scenario in which the greatest mean coefficient of variation and proportion of simulations with maximum weight greater than 100 was observed with unstabilised weights (coefficient of variation: 5.4, compared to 0.9 with stabilised weights; proportion of simulations with maximum weight over 100: 53.5%, compared to 7.3% with stabilised weights). Notably, in Scenarios 25–27 the mean coefficient of variation of the weights was always less than 1.5 and the proportion of simulations with maximum weight greater than 100 was always less than 14.0%, even with unstabilised weights.

### Impact of variation in weights on TSEipcw performance

**Fig. 6:**
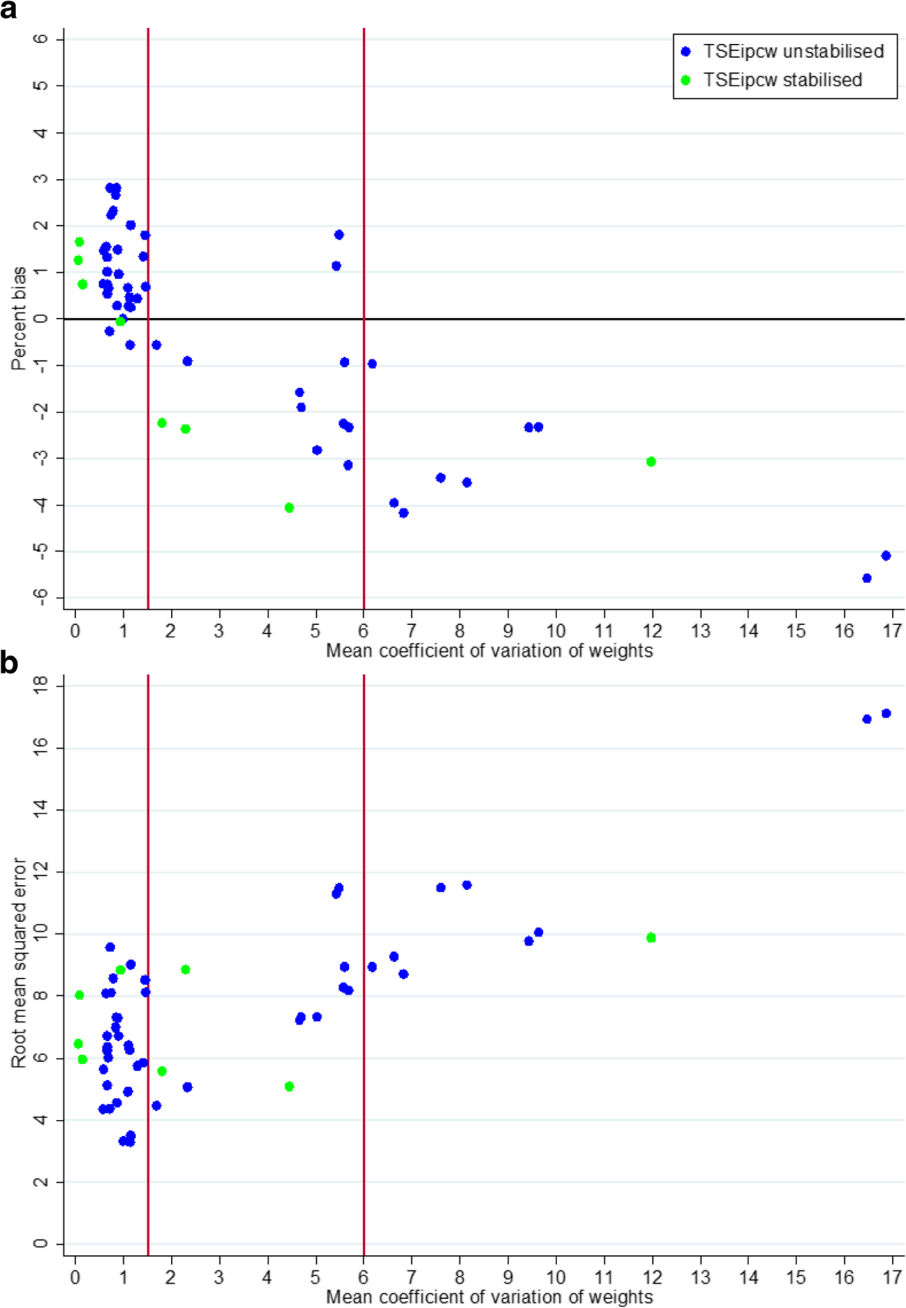
Impact of mean coefficient of variation in weights on TSEipcw performance: (**a**) percent bias; (**b**) Root mean squared error. Note: TSEipcw: two-stage estimation with inverse probability of censoring weights

Across all scenarios we consistently found that TSEipcw performed relatively well in scenarios where the mean coefficient of variation in the weights in the group in which switching was possible (i.e. amongst control group patients) was low, producing percentage bias similar to that produced by TSE and TSEnr. Figure 6 illustrates the relationship between the mean coefficient of variation in the inverse probability of censoring weights, percentage bias, and RMSE. When the mean coefficient of variation was less than 1.5, TSEipcw produced percentage bias that was generally positive and ranged between − 0.6 and 3.0%. As the mean coefficient of variation increased, there was a general trend towards more negative bias, with percentage bias ranging between approximately − 6.0 and 2.0%. However, whilst TSEipcw more often produced very low percentage bias when the mean coefficient of variation was less than 1.5, it still resulted in relatively high percentage bias (up to 3.0%) in some of these scenarios, and only resulted in percentage bias that was consistently worse when the mean coefficient of variation was greater than 6.0. There appears to be a positive correlation between the mean coefficient of variation in the inverse probability weights and RMSE. However, again, whilst TSEipcw more often resulted in very low levels of RMSE when the mean coefficient of variation was less than 1.5, it still resulted in relatively high RMSE in some of these scenarios, and RMSE only became consistently worse when the mean coefficient of variation was greater than 6.0.

There also appears to be a relationship between the size of the maximum weight and the performance of TSEipcw. In scenarios in which TSEipcw produced relatively high percentage bias and RMSE, the proportion of simulations that had maximum weights of greater than 100 and 1000 was high. To investigate this further we re-estimated percentage bias, empirical SE and RMSE for TSEipcw in all scenarios, only including simulations where the maximum weight was (i) ≤ 20; (ii) ≤100; (iii) ≤1000. Results are presented in Additional file 1: Appendix K. Often these analyses resulted in excluding large numbers of simulations and so they must be interpreted with caution – for instance, in Scenario 20, only 29 of the 1000 simulations had maximum weights less than 20; 226 had maximum weights less than 100, and; 565 had maximum weights less than 1000. Patterns in percentage bias remained similar to the overall results in each case. The empirical SE and RMSE associated with TSEipcw decreased when simulations with maximum weights of greater than 1000 and greater than 100 were excluded, especially in scenarios in which TSEipcw previously exhibited very high empirical SEs (such as Scenarios 20 and 36), but other changes were minimal.

## Discussion

Our study demonstrates the value of combining the two-stage adjustment method with IPCW to correct for informative censoring in counterfactual datasets derived when adjusting for treatment switching in clinical trials. We have demonstrated that the TSEipcw approach generally performs well when the estimated weights have a low coefficient of variation. Previous research has demonstrated that adjustment analyses that use re-censoring to address the informative censoring problem are likely to produce biased estimates of long-term control group mean survival time in situations where the survivor function is complex, with important changes in the hazard function over time [18]. For instance, when hazards decrease towards the end of trial follow-up (as simulated in the majority of scenarios in the present study), re-censoring is likely to cause this trend to be missed, resulting in under-estimates of long-term mean survival. In contrast, analyses that do not apply re-censoring are likely to produce over-estimates of long-term control group mean survival time (hence under-estimates of the treatment effect) [18]. Given this, using IPCW in combination with the TSE adjustment method represents a valid additional option for an analyst attempting to avoid the pitfalls of either re-censoring or not re-censoring. In the right circumstances, the TSEipcw approach can be used to enhance analyses presented to decision-makers who are interested in estimating long-term treatment effects in trials confounded by treatment switching.

The TSEipcw method produced lower percentage bias than the re-censoring and non-re-censoring options in 11 of the 48 scenarios, and in 9 of these it produced bias that was intermediate in relation to the negative bias produced by TSE and the positive bias produced by TSEnr. TSEipcw was most likely to produce lower bias than TSE and TSEnr when the treatment effect was high – in these scenarios re-censoring results in a large amount of lost information, whilst not re-censoring results in more important informative censoring bias. In several other scenarios TSEipcw produced percentage bias that was of a similar size to that produced by TSE and TSEnr. It may be argued that TSEipcw has theoretical advantages over TSE and TSEnr, given the HTA context where the objective is to estimate long-term survival. Re-censoring is sub-optimal because it leads to a loss of longer-term information, whilst a failure to re-censor means that results are prone to informative censoring bias. TSEipcw does not involve a loss of information and protects against informative censoring bias, provided the no unmeasured confounding assumption is satisfied.

However, we found that TSEipcw has important limitations in several of the scenarios that we simulated. TSEipcw was the worst performing adjustment method in scenarios where the mean coefficient of variation of the weights was high and where a large proportion of simulations had very high weights (greater than 100). Our results suggest that if the coefficient of variation is less than approximately 1.5, TSEipcw is likely to perform consistently and well. When the coefficient of variation is higher, the method becomes more inconsistent and prone to higher levels of bias and RMSE, and should not be relied upon. Notably, this coefficient of variation is measured only across patients in the control group, as these were the only patients to which weights were applied in our study. The coefficient of variation would be substantially reduced if we included experimental group patients in its estimation, because all these patients received weights of 1 across all time-periods. Our findings are in line with previous research, which has shown that IPCW methods become prone to substantial error when observations are assigned extreme weights [11, 12, 33]. In this study, high weight variation was often observed in scenarios with low disease severity (and thus a high censoring proportion), but this was not always the case. In fact, high weight variation was only observed when disease severity was low and the simulated disease mechanism was such that a substantial proportion of patients censored at the end of the study had not experienced disease progression. Importantly, we conclude that TSEipcw will not necessarily perform poorly in the presence of a high censoring proportion – rather, it becomes prone to bias when the coefficient of variation in the weights is high, irrespective of the reason for the large range in the estimated weights.

We also found that the performance of TSEipcw was relatively poor in scenarios with a high proportion of simulations that resulted in very high maximum weights. However, we are unable to conclude what size maximum weight is “too large” – in scenarios in which TSEipcw performed poorly it generally continued to perform poorly even when simulations with maximum weights of greater than 20 were excluded. In practice, choosing an optimal weighting model is not straightforward. Previous research has shown that changing the model specification can result in drastically different weights [46]. It therefore follows that various model specifications should be explored. Weight truncation could be considered, though this should be used with caution as it may re-introduce bias [46].

Given our suggestion that replacing re-censoring with IPCW represents a valid option, the limitations of this approach should be considered. In particular, in this context IPCW is applied to a counterfactual dataset. IPCW analyses rely upon the ‘no unmeasured confounding’ assumption, and therefore require information on baseline and time-dependent covariates that are prognostic for survival and predict censoring. In the context where TSE is used to adjust survival times to account for treatment switching, and then IPCW is applied to the resulting counterfactual dataset, observations on time-varying characteristics (such as biomarker values) beyond the point of switching are of questionable use because they may have been affected by the treatment switch. In our simulations, we had perfect information on such variables, because we simulated their values prior to applying treatment switching – reflecting a situation where time-varying values beyond the switch point could be perfectly predicted, adjusted for switching. In reality, structural mean models may be required to estimate these values, further complicating the analysis. To test the sensitivity of the TSEipcw method to violations of the no unmeasured confounding assumption, we included a version of the TSEipcw analysis that assumed that no information was available on the time-varying biomarker, and hence the only time-varying information included in the weighting model was progression time – which represented time-varying information that was not affected by treatment switching because switching only happened after disease progression. This approach performed almost identically to the approach that incorporated full information on the time-varying biomarker, because there was a strong association between the biomarker and progression time in our simulated datasets. This may not always be the case – in practice, clinical expert knowledge and causal pathways should be examined to identify whether values of time-dependent variables need to be predicted beyond the switching time-point in order to adequately satisfy the no unmeasured confounding assumption.

A further limitation of the IPCW approach in this context is that random entry into the study combined with a calendar-date study end time-point may result in very low numbers at risk at the end of the study follow-up period, resulting in large weights for the remaining observations. We did not attempt to address this issue in our simulation study, and TSEipcw still performed well in several scenarios. In reality a small amount of re-censoring could be applied to avoid this problem if it was identified as the reason for obtaining large weights. This would involve the loss of some longer-term information, but substantially less than under the full re-censoring approach.

Our study has limitations. As previously described, a simulation study can never be exhaustive in relation to the scenarios investigated, alternative methods could be used to generate the simulated datasets, and alternative endpoints could be considered important for different contexts [18]. We believe that our simulation mechanism allowed us to generate realistic datasets, and our endpoint of restricted mean survival is relevant given our focus on analyses used to inform HTA analyses, whilst avoiding the problems associated with extrapolation that would have arisen had we have chosen to focus on even longer term (perhaps lifetime) mean survival.

It may be considered to be a limitation that we would not expect any of the adjustment methods to work perfectly in our simulated scenarios, because underlying survival times were simulated using a mixture Weibull model incorporating a time-dependent covariate, whereas we used flexible parametric spline-based models to estimate the RMST associated with each method adjustment. We included the correct variables within the adjustment models and therefore the no unmeasured confounders assumption held, but the underlying survival models were different. This was intentional because in reality true underlying survival models are unknown. Common practice in HTA is to use a flexible parametric model if the observed hazards are complex (i.e. with turning points) [41, 42] and therefore we took this approach in this study.

Perhaps the most important limitation of our study is that in most scenarios we simulated a complex hazard function which first increased and then decreased within the study period. This is a key driver of the results associated with the application of TSE that applied re-censoring, because the associated loss of longer-term information was important – an important change in the trend in the hazard was missed when longer-term information was lost, resulting in biased estimates of mean survival time restricted to the end of the simulated trial follow-up period. As previously stated, we believe that the hazard and survival functions that we simulated are realistic, but in some cases such trends in the hazard may not be observed; turning points or changes in slope of the hazard function may occur beyond the end of the trial, or may not occur at all. In such cases, re-censoring should not result in bias due to lost longer-term trial information.

It is also relevant to note that we only incorporated the two-stage adjustment method in this study – we did not include the rank preserving structural failure time model (RPSFTM) [47]. The RPSFTM has the same problems associated with censoring as the two-stage method, and therefore combining the RPSFTM with IPCW represents an alternative to re-censoring in the same way that it does for TSE. We did not assess RPSFTM combined with IPCW because doing so is not straightforward: inverse probability of censoring weights would need to be estimated for every value of *ψ* included within the g-estimation process. This would be computationally intensive, especially within a simulation study where thousands of analyses are conducted. Therefore, we focussed our investigation on the two-stage method. In theory, we would expect IPCW to perform similarly when combined with RPSFTM as when combined with TSE, because in both cases it is simply being applied to a counterfactual dataset in which adjustments have been made for treatment switching.

## Conclusions

Incorporating IPCW within two-stage adjustment analyses represents a credible alternative to re-censoring or not re-censoring, provided that estimated weights are not extreme in size and have relatively low variation. The characteristics of the trial and treatment under investigation and the objectives of the research are important to consider: if the objective is to estimate long-term treatment effects and there is likely to be important changes in the hazard function and the treatment effect over time, re-censored analyses are prone to bias due to lost longer-term information, and non-re-censored analyses are prone to informative censoring bias. These problems are particularly important when the treatment effect is high. Provided the no unmeasured confounding assumption is reasonable and that estimated weights have low variation, we recommend presenting TSEipcw alongside adjustment analyses with and without re-censoring to provide decision-makers with enhanced information on the range in which the true treatment effect is likely to lie. If weights have high variation TSEipcw should not be relied upon, and we recommend considering the characteristics of the trial and the results of previous research [18] to help determine whether re-censoring or not re-censoring represents the approach likely to result in least bias.

## Additional files

Additional file 1: **Appendix A**: Simulation study code. **Appendix B**: Simulation analysis ado code for ‘itt’ and ‘tswnew’. **Appendix C**: Treatment switching probabilities. **Appendix D**: Scenario parameter values. **Appendix E**: Scenario settings. **Appendix F**: Overview of simulation scenarios.**Appendix G**: Percentage bias across all scenarios. **Appendix H**: Empirical standard error of percentage bias across all scenarios.**Appendix I**: Root mean squared error of percentage bias across all scenarios. **Appendix J**: TSEipcw with stabilised and unstabilised weights. **Appendix K**: Percentage bias, empirical standard error and root mean squared error when simulations with high maximum weights are excluded (DOCX 196 kb)

## Abbreviations

AF: Acceleration factor
CI: Confidence interval
HR: Hazard ratio
HTA: Health technology assessment
IPCW: Inverse probability of censoring weights
ITT: Intention to treat
MSM: Marginal structural model
RCT: Randomised controlled trial
RMSE: Root mean squared error
RMST: Restricted mean survival time
RPSFTM: Rank preserving structural failure time model
TSE: Two-stage estimation
TSEipcw: Two-stage estimation combined with inverse probability of censoring weights
TSEnr: Two-stage estimation without re-censoring
