# Supplementary material for: Two-stage estimation to adjust for treatment switching in randomised trials: a simulation study investigating the use of inverse probability weighting instead of re-censoring
Source: BMC Med Res Methodol. 2019 Mar 29;19:69. doi: 10.1186/s12874-019-0709-9 (PMC6444622; doi:10.1186/s12874-019-0709-9)
Supplement: Supplementary file 1 — Appendix A: Simulation study code. Appendix B: Simulation analysis ado code for ‘itt’ and ‘tswnew’. Appendix C: Treatment switching probabilities. Appendix D: Scenario parameter values. Appendix E: Scenario settings. Appendix F: Overview of simulation scenarios.Appendix G: Percentage bias across all scenarios. Appendix H: Empirical standard error of percentage bias across all scenarios.Appendix I: Root mean squared error of percentage bias across all scenarios. Appendix J: TSEipcw with stabilised and unstabilised weights. Appendix K: Percentage bias, empirical standard error and root mean squared error when simulations with high maximum weights are excluded (DOCX 196 kb) [file 12874_2019_709_MOESM1_ESM.docx]

**APPENDICES**

# Appendix A: Simulation study code

# *** NOTE BEFORE RUNNING, SET SEED 123456789 ***

# cd "C:\simulationsv7arecensv2"

# capture program drop simstudyv1

# program define simstudyv1, rclass

# version 13.1

# syntax [, obs(int 500) bprog(real 0.5) betain(real 20) ///

# betasl(real 0.04) alphat(real 0.0) bprogin(real 2.5) trtlghr(real -0.22) bprogsl(real 0.3) ///

# tde(real 0.00) beta2(real -0.02) admin(real 548) logitcea31(real 0.01) ///

# logitcea32(real 0.03) logitcea33(real 0.11) logcea21(real 2) logcea22(real 2) ///

# logcea23(real 2) logcea11(real 3.5) logcea12(real 3.5) logcea13(real 3.5) ///

# logxo2(real 0.8) logxo3(real 0.5) xomult(real 1.13000518614461) ///

# LAMBDA1(real 0.00001) ///

# LAMBDA2(real 0.00001) ///

# GAMMA1(real 1.8) ///

# GAMMA2(real 0.8) ///

# Pmix(real 1.0) ///

# ]

# clear

# adopath ++ "C:\ado\plus\s\survsim"

# *** 1. GENERATE SURVIVAL TIMES ***

# clear

# pr drop _all

# set obs `obs'

# gen trtrand = rbinomial(1,0.66)

# gen bprog = rbinomial(1,`bprog')

# gen b0 = rnormal(`betain',1)

# gen admin = `admin'

#

# survsim timeOS event, hazard( ///

# (`lambda1':*`gamma1':*`pmix':*#t:^(`gamma1':-1):*exp(-`lambda1':*#t:^`gamma1') :+ ///

# `lambda2':*`gamma2':*(1:-`pmix'):*#t:^(`gamma2':-1):* ///

# exp(-`lambda2':*#t:^`gamma2') ):/(`pmix':*exp(-`lambda1':*#t:^`gamma1') ///

# :+ (1:-`pmix'):*exp(-`lambda2':*#t:^`gamma2')) ///

# :* ///

# exp( `alphat' :* (b0 :+ `betasl' :*#t :+ `beta2':*#t :*trtrand :+ bprog:*`bprogin') ) ) ///

# cov(trtrand `trtlghr' bprog `bprogsl') tde(trtrand `tde') maxtime(3000) nodes(100)

#

# gen id = _n

# rename event dead

# replace timeOS = round(timeOS)

# replace timeOS=1 if timeOS==0

# ***create random entry day for control group patients***

# gen entry = floor((183-0+1)*runiform() + 0) if trtrand==0

# replace entry = 0 if trtrand==1

# replace admin = admin - entry

# *** 2. CALCULATE HR AND RMST WITH NO SWITCHING ***

# preserve

# replace dead = 0 if timeOS>admin

# replace timeOS = admin if timeOS>admin

# stset timeOS, failure(dead) id(id)

# stci if trtrand==0, rmean

# return scalar simtrueconstcimean = r(rmean)

# stci if trtrand==1, rmean

# return scalar simtrueexpstcimean = r(rmean)

#

# capture stpm2 trtrand bprog, scale(h) df(4) lininit iterate(200)

# if e(converged)==. | e(converged)==0 {

# capture stpm2 trtrand bprog, scale(h) df(3) lininit iterate(200)

# if e(converged)==. | e(converged)==0 {

# capture stpm2 trtrand bprog, scale(h) df(2) lininit iterate(200)

# if e(converged)==. | e(converged)==0 {

# capture stpm2 trtrand bprog, scale(h) df(1) lininit iterate(200)

# }

# }

# }

# return scalar simtrue_fpmwbprog_hr = exp(_b[trtrand])

# gen conv1 = e(converged)

# summ conv1

# return scalar simtrue_fpmwbrog_conv = r(mean)

#

# capture stpm2 trtrand, scale(h) df(4) lininit iterate(200)

# if e(converged)==. | e(converged)==0 {

# capture stpm2 trtrand, scale(h) df(3) lininit iterate(200)

# if e(converged)==. | e(converged)==0 {

# capture stpm2 trtrand, scale(h) df(2) lininit iterate(200)

# if e(converged)==. | e(converged)==0 {

# capture stpm2 trtrand, scale(h) df(1) lininit iterate(200)

# }

# }

# }

# return scalar simtrue_fpm_hr = exp(_b[trtrand])

# gen conv2 = e(converged)

# summ conv2

# return scalar simtrue_fpm_conv = r(mean)

# streg trtrand bprog, dist(weibull) iterate(200)

# return scalar simtrue_weibwbprog_hr = exp(_b[trtrand])

# gen conv3 = e(converged)

# summ conv3

# return scalar simtrue_weibwbprog_conv = r(mean)

# streg trtrand, dist(weibull) iterate(200)

# return scalar simtrue_weib_hr = exp(_b[trtrand])

# gen conv4 = e(converged)

# summ conv4

# return scalar simtrue_weib_conv = r(mean)

#

# capture stpm2 if trtrand==1, scale(h) df(4) lininit iterate(200)

# if e(converged)==. | e(converged)==0 {

# capture stpm2 if trtrand==1, scale(h) df(3) lininit iterate(200)

# if e(converged)==. | e(converged)==0 {

# capture stpm2 if trtrand==1, scale(h) df(2) lininit iterate(200)

# if e(converged)==. | e(converged)==0 {

# capture stpm2 if trtrand==1, scale(h) df(1) lininit iterate(200)

# }

# }

# }

# gen conv5 = e(converged)

# predict s, survival

# keep id trtrand dead timeOS s conv5

# expand 2 if id==1

# gen id2=_n

# egen id3=max(id2)

# replace timeOS=0 if id2==id3

# replace s = 1 if id2==id3

# replace trtrand=1 if id2==id3

# drop if trtrand==0

# sort timeOS

# gen auc=0

# replace auc =(timeOS-timeOS[_n-1])*((s+s[_n-1])/2)

# egen auc1=total(auc)

# summ auc1

# return scalar simtruefpmexpauc=r(mean)

# summ conv5

# return scalar simtrue_fpmexp_conv = r(mean)

#

# restore

# preserve

# replace dead = 0 if timeOS>admin

# replace timeOS = admin if timeOS>admin

# stset timeOS, failure(dead) id(id)

# capture stpm2 if trtrand==0, scale(h) df(4) lininit iterate(200)

# if e(converged)==. | e(converged)==0 {

# capture stpm2 if trtrand==0, scale(h) df(3) lininit iterate(200)

# if e(converged)==. | e(converged)==0 {

# capture stpm2 if trtrand==0, scale(h) df(2) lininit iterate(200)

# if e(converged)==. | e(converged)==0 {

# capture stpm2 if trtrand==0, scale(h) df(1) lininit iterate(200)

# }

# }

# }

# gen conv6 = e(converged)

# predict s, survival

# keep id trtrand dead timeOS s conv6

# expand 2 if id==1

# gen id2=_n

# egen id3=max(id2)

# replace timeOS=0 if id2==id3

# replace s = 1 if id2==id3

# replace trtrand=0 if id2==id3

# drop if trtrand==1

# sort timeOS

# gen auc=0

# replace auc =(timeOS-timeOS[_n-1])*((s+s[_n-1])/2)

# egen auc1=total(auc)

# summ auc1

# return scalar simtruefpmconauc=r(mean)

# summ conv6

# return scalar simtrue_fpmcon_conv = r(mean)

#

# restore

#

# *** 3. GENERATE PROGRESSION TIMES ***

# stset timeOS, failure(dead) id(id)

# gen timePFS = round(timeOS*rbeta(5,10))

#

# ***calculate when PFS is observed, as can only occur at scheduled appointments. Hence need to split data here***

# stsplit timeOS2, every(21)

# sort id

# by id: gen PFSobsind=1 if timePFS<timeOS2

# by id: replace PFSobsind=. if timePFS>admin

# by id: gen timePFSobst=timeOS2 if PFSobsind==1

# by id: egen timePFSobs=min(timePFSobst)

# ***Note: some patients who have a PFStime and who die will not have PFS observed, because progression ***and death occur in the same time interval. These patients have progressed==0***

# by id: egen timeOSreal=max(timeOS)

# by id: gen progressed = 1 if timePFSobs!=.

# by id: replace progressed = 0 if timePFSobs==.

# by id: replace timePFSobs = timeOSreal if progressed==0

# by id: replace timePFSobs = admin if (timeOSreal > admin & progressed==0)

# by id: replace timePFSobs = admin if (timePFSobs > admin & progressed==1)

# by id: replace progressed = 0 if timePFSobs==admin

# ***now have this, can collapse the dataset again.***

# collapse (max) trtrand bprog timeOS timePFS timePFSobs b0 admin progressed dead, by(id)

#

# *** 4. GENERATE OSBERVED BIOMARKER (CEA) VALUES ***

# ***below we estimate underlying cea levels at the time of disease progression, and at the two following ***consultations.***

# ***rnormal reflects a random error in the cea term***

# ***note we only need to estimate actual cea values for the control group since it is only these that crossover. ***Therefore we do not need to include a treatment term here***

# gen cea1 = b0 + `betasl'*(timePFSobs) + `bprogin'*bprog + rnormal(0,1) if trtrand==0 & timeOS>timePFSobs & /// progressed==1

# gen cea2 = b0 + `betasl'*(timePFSobs+(21)) + `bprogin'*bprog + rnormal(0,1) if trtrand==0 & timeOS > timePFSobs+(21) /// & progressed==1

# gen cea3 = b0 + `betasl'*(timePFSobs+(42)) + `bprogin'*bprog + rnormal(0,1) if trtrand==0 & timeOS > timePFSobs+(42) /// & progressed==1

# ***Cut underlying cea into three groups based on 33.3% centiles so that can allow probability of switch to depend upon the ***category of cea level***

# egen cea1grp = cut(cea1), group(3)

# egen cea2grp = cut(cea2), group(3)

# egen cea3grp = cut(cea3), group(3)

#

# *** 5. GENERATE SWITCH PROBABILITIES AND TIMES ***

# ***Cut timePFSobs in the control group into three groups so that can allow probability of switch to depend on these ***categories***

# gen timePFScontrolobs = timePFSobs if trtrand==0 & progressed==1

# egen timePFSobsgrp = cut(timePFScontrolobs), group(3)

# drop timePFScontrolobs

#

# ***base switch probability on CEA group and timePFSobs group***

# gen p1 = (invlogit(logit(`logitcea31') + log(`logcea21')*(cea1grp==1) + log(`logcea11')*(cea1grp==2))) if /// timePFSobsgrp==0

# replace p1 = (invlogit(logit(`logitcea32') + log(`logcea22')*(cea1grp==1) + log(`logcea12')*(cea1grp==2))) /// if timePFSobsgrp==1

# replace p1 = (invlogit(logit(`logitcea33') + log(`logcea23')*(cea1grp==1) + log(`logcea13')*(cea1grp==2))) /// if timePFSobsgrp==2

# gen xo1 = rbinomial(1,p1) if trtrand==0 & timeOS>timePFSobs & progressed==1

# gen xotime = timePFSobs if xo1==1

# ***switch becomes more unlikely in the second and third consultations after progression***

# gen p2 = (invlogit(logit(`logitcea31') + log(`logcea21')*(cea2grp==1) + log(`logcea11')*(cea2grp==2) /// +log(`logxo2'))) if timePFSobsgrp==0

# replace p2 = (invlogit(logit(`logitcea32') + log(`logcea22')*(cea2grp==1) + log(`logcea12')*(cea2grp==2) /// +log(`logxo2'))) if timePFSobsgrp==1

# replace p2 = (invlogit(logit(`logitcea33') + log(`logcea23')*(cea2grp==1) + log(`logcea13')*(cea2grp==2) /// +log(`logxo2'))) if timePFSobsgrp==2

# gen xo2 = rbinomial(1,p2) if trtrand==0 & xo1 == 0 & timeOS > timePFSobs+(21) & progressed==1

# replace xotime = timePFSobs +(21) if xo2==1

# gen p3 = (invlogit(logit(`logitcea31') + log(`logcea21')*(cea3grp==1) + log(`logcea11')*(cea3grp==2) /// +log(`logxo3'))) if timePFSobsgrp==0

# replace p3 = (invlogit(logit(`logitcea32') + log(`logcea22')*(cea3grp==1) + log(`logcea12')*(cea3grp==2) /// +log(`logxo3'))) if timePFSobsgrp==1

# replace p3 = (invlogit(logit(`logitcea33') + log(`logcea23')*(cea3grp==1) + log(`logcea13')*(cea3grp==2) /// +log(`logxo3'))) if timePFSobsgrp==2

# gen xo3 = rbinomial(1,p3) if trtrand==0 & xo1 == 0 & xo2==0 & timeOS > timePFSobs+(42) & progressed==1

# replace xotime = timePFSobs +(42) if xo3==1

#

# gen xo= 1 if (xo1==1 | xo2==1 | xo3==1)

#

# *** 6. GENERATE NEW SURVIVAL TIMES IN SWITCHERS ***

# gen xoOSgainobs = timeOS-xotime if xo==1

# replace xoOSgainobs = round(xoOSgainobs*`xomult')

# gen timeOS2 = cond(xo==1, xoOSgainobs + xotime,timeOS)

#

# *** 7. INCORPORATE CENSORING ***

# gen died=0

# replace died = 1 if timeOS2<=admin & dead==1

# drop dead

# replace timeOS2=admin if timeOS2>admin

# replace xoOSgainobs=. if xotime>admin

# replace xo=. if xotime>=admin

# replace xotime=. if xotime>=admin

#

# *** 8. CREATE A PANEL OF DATA ***

# stset timeOS2, failure(died) id(id)

# stsplit timeOS3, every(21)

# sort id

# by id: gen obsno=_n

# tsset id obsno

# **generate observed CEA for each observation which reflect***

# gen obscea = b0 + `betasl'*(_t0) + `bprogin'*bprog +`beta2'*trtrand*(_t0) + rnormal(0,1)

# replace died=0 if died==.

# sort id

# by id: replace b0 = b0 + (`bprogin'*bprog)

# by id: replace obscea = b0 if _n==1

# ***generate time-dependent switch indicator***

# gen xoti=0

# replace xoti=1 if xo==1 & (timeOS3)>=(xotime)

# ***set obscea = cea1, cea2 and cea3 where relevant***

# by id: gen xoconsult1=1 if (timeOS3)==(timePFSobs) & trtrand==0

# by id: gen xoconsult2=1 if xoconsult1[_n-1] ==1 & trtrand==0

# by id: gen xoconsult3=1 if xoconsult2[_n-1] ==1 & trtrand==0

# by id: replace obscea = cea1 if xoconsult1==1

# by id: replace obscea = cea2 if xoconsult2==1 & cea2!=.

# by id: replace obscea = cea3 if xoconsult3==1 & cea3!=.

# by id: gen finalobs = 0

# by id: replace finalobs = 1 if _n==_N

# gen cens=0

# replace cens=1 if finalobs==1 & _t==admin & died==0

# replace died=. if cens==1

#

# *** 9. RECORD SOME DIAGNOSTICS (SWITCH PROPORTION, CENSORING NUMBER ***

# by id: egen xoind=max(xo)

# replace xoind=0 if xoind==.

# by id: egen xoprop=max(timeOS3)

# replace xoprop=xoprop/(21)

# replace xoprop=xoprop+1

# replace xoprop=xoind/xoprop

# summ xoprop if trtrand==0

# return scalar xo_number=r(mean)*r(N)

# summ cens

# return scalar cens_number=r(mean)*r(N)

# summ cens if trtrand==0

# return scalar cens_number_con=r(mean)*r(N)

#

# *** 10. RUN ANALYSES ***

#

# itt

# return scalar prog_number=r(prog_number)

# return scalar prog_number_con=r(prog_number_con)

# return scalar con_number=r(con_number)

# return scalar cens_number_xo=r(cens_number_xo)

# return scalar itt_auc_con = r(itt_auc_con)

# return scalar itt_auc_con_LB = r(itt_auc_con_LB)

# return scalar itt_auc_con_UB = r(itt_auc_con_UB)

# return scalar itt_auc_con_conv = r(itt_auc_con_conv)

# return scalar itt_cox_hr = r(itt_cox_hr)

# return scalar itt_cox_hr_SE = r(itt_cox_hr_SE)

# return scalar itt_cox_hr_LB = r(itt_cox_hr_LB)

# return scalar itt_cox_hr_UB = r(itt_cox_hr_UB)

# return scalar itt_fpm_hr = r(itt_fpm_hr)

# return scalar itt_fpm_hr_SE = r(itt_fpm_hr_SE)

# return scalar itt_fpm_hr_LB = r(itt_fpm_hr_LB)

# return scalar itt_fpm_hr_UB = r(itt_fpm_hr_UB)

# return scalar itt_fpm_hr_conv = r(itt_fpm_hr_conv)

# return scalar exp_fpm_conv = r(exp_fpm_conv)

# tswnew

# return scalar weib2m_weib_af = r(weib2m_weib_af)

# return scalar weib2m_weib_af_SE = r(weib2m_weib_af_SE)

# return scalar weib2m_weib_af_LB = r(weib2m_weib_af_LB)

# return scalar weib2m_weib_af_UB = r(weib2m_weib_af_UB)

# return scalar weib2m_weib_af_conv = r(weib2m_weib_af_conv)

# return scalar weib2m_adj_auc_con = r(weib2m_adj_auc_con)

# return scalar weib2m_adj_fpm_con_conv = r(weib2m_adj_fpm_con_conv)

# return scalar weib2m_adj_fpm_hr = r(weib2m_adj_fpm_hr)

# return scalar weib2m_adj_fpm_hr_SE = r(weib2m_adj_fpm_hr_SE)

# return scalar weib2m_adj_fpm_hr_LB = r(weib2m_adj_fpm_hr_LB)

# return scalar weib2m_adj_fpm_hr_UB = r(weib2m_adj_fpm_hr_UB)

# return scalar weib2m_adj_fpm_conv = r(weib2m_adj_fpm_conv)

# return scalar tsw_ipcw1_conv = r(tsw_ipcw1_conv)

# return scalar tsw_ipcw1_omit = r(tsw_ipcw1_omit)

# return scalar tsw_ipcw1_cds = r(tsw_ipcw1_cds)

# return scalar tsw_ipcw1_cdf = r(tsw_ipcw1_cdf)

# return scalar tsw_ipcw3_conv = r(tsw_ipcw3_conv)

# return scalar tsw_ipcw3_omit = r(tsw_ipcw3_omit)

# return scalar tsw_ipcw3_cds = r(tsw_ipcw3_cds)

# return scalar tsw_ipcw3_cdf = r(tsw_ipcw3_cdf)

# return scalar tsw_ipcw_cox_hr = r(tsw_ipcw_cox_hr)

# return scalar tsw_ipcw_cox_hr_SE = r(tsw_ipcw_cox_hr_SE)

# return scalar tsw_ipcw_cox_hr_LB = r(tsw_ipcw_cox_hr_LB)

# return scalar tsw_ipcw_cox_hr_UB = r(tsw_ipcw_cox_hr_UB)

# return scalar tsw_ipcw_weight_min = r(tsw_ipcw_weight_min)

# return scalar tsw_ipcw_weight_max = r(tsw_ipcw_weight_max)

# return scalar tsw_ipcw_weight_mean = r(tsw_ipcw_weight_mean)

# return scalar tsw_ipcw_weight_sd = r(tsw_ipcw_weight_sd)

# return scalar tsw_ipcw_weight_cv = r(tsw_ipcw_weight_cv)

# return scalar tsw_ipcw_adj_auc_con = r(tsw_ipcw_adj_auc_con)

# return scalar tsw_ipcw_adj_auc_conv = r(tsw_ipcw_adj_auc_conv)

# return scalar weib2m_adj_aucnr_con = r(weib2m_adj_aucnr_con)

# return scalar weib2m_adj_fpm_con_convnr = r(weib2m_adj_fpm_con_convnr)

# return scalar weib2m_adj_fpm_hrnr = r(weib2m_adj_fpm_hrnr)

# return scalar weib2m_adj_fpm_hrnr_SE = r(weib2m_adj_fpm_hrnr_SE)

# return scalar weib2m_adj_fpm_hrnr_LB = r(weib2m_adj_fpm_hrnr_LB)

# return scalar weib2m_adj_fpm_hrnr_UB = r(weib2m_adj_fpm_hrnr_UB)

# return scalar weib2m_adj_fpm_convnr = r(weib2m_adj_fpm_convnr)

# end

# simulate xo_number = r(xo_number) ///

# cens_number = r(cens_number) ///

# cens_number_con = r(cens_number_con) ///

# prog_number = r(prog_number) ///

# prog_number_con = r(prog_number_con) ///

# con_number = r(con_number) ///

# cens_number_xo = r(cens_number_xo) ///

# simtrueconstcimean = r(simtrueconstcimean) ///

# simtrueexpstcimean = r(simtrueexpstcimean) ///

# simtrue_fpmwbprog_hr = r(simtrue_fpmwbprog_hr) ///

# simtrue_fpm_hr = r(simtrue_fpm_hr) ///

# simtruefpmexpauc=r(simtruefpmexpauc) ///

# simtruefpmconauc=r(simtruefpmconauc) ///

# simtrue_weibwbprog_hr = r(simtrue_weibwbprog_hr) ///

# simtrue_weib_hr = r(simtrue_weib_hr) ///

# simtrue_fpmwbrog_conv = r(simtrue_fpmwbrog_conv) ///

# simtrue_fpm_conv = r(simtrue_fpm_conv) ///

# simtrue_weibwbprog_conv = r(simtrue_weibwbprog_conv) ///

# simtrue_weib_conv = r(simtrue_weib_conv) ///

# simtrue_fpmexp_conv = r(simtrue_fpmexp_conv) ///

# simtrue_fpmcon_conv = r(simtrue_fpmcon_conv) ///

# itt_auc_con = r(itt_auc_con) ///

# itt_auc_con_LB = r(itt_auc_con_LB) ///

# itt_auc_con_UB = r(itt_auc_con_UB) ///

# itt_auc_con_conv = r(itt_auc_con_conv) ///

# itt_cox_hr = r(itt_cox_hr) ///

# itt_cox_hr_SE = r(itt_cox_hr_SE) ///

# itt_cox_hr_LB = r(itt_cox_hr_LB) ///

# itt_cox_hr_UB = r(itt_cox_hr_UB) ///

# itt_fpm_hr = r(itt_fpm_hr) ///

# itt_fpm_hr_SE = r(itt_fpm_hr_SE) ///

# itt_fpm_hr_LB = r(itt_fpm_hr_LB) ///

# itt_fpm_hr_UB = r(itt_fpm_hr_UB) ///

# itt_fpm_hr_conv = r(itt_fpm_hr_conv) ///

# exp_fpm_conv = r(exp_fpm_conv) ///

# weib2m_weib_af = r(weib2m_weib_af) ///

# weib2m_weib_af_SE = r(weib2m_weib_af_SE) ///

# weib2m_weib_af_LB = r(weib2m_weib_af_LB) ///

# weib2m_weib_af_UB = r(weib2m_weib_af_UB) ///

# weib2m_weib_af_conv = r(weib2m_weib_af_conv) ///

# weib2m_adj_auc_con = r(weib2m_adj_auc_con) ///

# weib2m_adj_fpm_con_conv = r(weib2m_adj_fpm_con_conv) ///

# weib2m_adj_fpm_hr = r(weib2m_adj_fpm_hr) ///

# weib2m_adj_fpm_hr_SE = r(weib2m_adj_fpm_hr_SE) ///

# weib2m_adj_fpm_hr_LB = r(weib2m_adj_fpm_hr_LB) ///

# weib2m_adj_fpm_hr_UB = r(weib2m_adj_fpm_hr_UB) ///

# weib2m_adj_fpm_conv = r(weib2m_adj_fpm_conv) ///

# tsw_ipcw1_conv = r(tsw_ipcw1_conv) ///

# tsw_ipcw1_omit = r(tsw_ipcw1_omit) ///

# tsw_ipcw1_cds = r(tsw_ipcw1_cds) ///

# tsw_ipcw1_cdf = r(tsw_ipcw1_cdf) ///

# tsw_ipcw3_conv = r(tsw_ipcw3_conv) ///

# tsw_ipcw3_omit = r(tsw_ipcw3_omit) ///

# tsw_ipcw3_cds = r(tsw_ipcw3_cds) ///

# tsw_ipcw3_cdf = r(tsw_ipcw3_cdf) ///

# tsw_ipcw_cox_hr = r(tsw_ipcw_cox_hr) ///

# tsw_ipcw_cox_hr_SE = r(tsw_ipcw_cox_hr_SE) ///

# tsw_ipcw_cox_hr_LB = r(tsw_ipcw_cox_hr_LB) ///

# tsw_ipcw_cox_hr_UB = r(tsw_ipcw_cox_hr_UB) ///

# tsw_ipcw_weight_min = r(tsw_ipcw_weight_min) ///

# tsw_ipcw_weight_max = r(tsw_ipcw_weight_max) ///

# tsw_ipcw_weight_mean = r(tsw_ipcw_weight_mean) ///

# tsw_ipcw_weight_sd = r(tsw_ipcw_weight_sd) ///

# tsw_ipcw_weight_cv = r(tsw_ipcw_weight_cv) ///

# tsw_ipcw_adj_auc_con = r(tsw_ipcw_adj_auc_con) ///

# tsw_ipcw_adj_auc_conv = r(tsw_ipcw_adj_auc_conv) ///

# weib2m_adj_aucnr_con = r(weib2m_adj_aucnr_con) ///

# weib2m_adj_fpm_con_convnr = r(weib2m_adj_fpm_con_convnr) ///

# weib2m_adj_fpm_hrnr = r(weib2m_adj_fpm_hrnr) ///

# weib2m_adj_fpm_hrnr_SE = r(weib2m_adj_fpm_hrnr_SE) ///

# weib2m_adj_fpm_hrnr_LB = r(weib2m_adj_fpm_hrnr_LB) ///

# weib2m_adj_fpm_hrnr_UB = r(weib2m_adj_fpm_hrnr_UB) ///

# weib2m_adj_fpm_convnr = r(weib2m_adj_fpm_convnr), ///

# reps(1000) saving(simulationv1): simstudyv1, obs(500) bprog(0.5) betain(20) betasl(0.04) alphat(0.0) /// bprogin(2.5) trtlghr(-0.22) bprogsl(0.3) tde(0.00) beta2(-0.02) admin(548) logitcea31(0.01) logitcea32(0.03) /// logitcea33(0.11) logcea21(2) logcea22(2) logcea23(2) logcea11(3.5) logcea12(3.5) logcea13(3.5) /// logxo2(0.8) logxo3(0.5) xomult(1.13000518614461)

# Appendix B: Simulation analysis ado code for ‘itt’ and ‘tswnew’

# program define itt, rclass

# version 11

# *** 1. ITT ANALYSIS ***

# ***Cox Proportional Hazards Analysis***

# stcox trtrand

# return scalar itt_cox_hr = exp(_b[trtrand])

# return scalar itt_cox_hr_SE = exp(_b[trtrand])*_se[trtrand]

# return scalar itt_cox_hr_LB = exp((_b[trtrand])-(1.96*_se[trtrand]))

# return scalar itt_cox_hr_UB = exp((_b[trtrand])+(1.96*_se[trtrand]))

# ***first some diagnostics...***

# preserve

# collapse (max) trtrand bprog b0 timeOS2 xoOSgainobs xotime died cens timePFSobs admin progressed xo, by(id)

# summ progressed

# return scalar prog_number=r(mean)*r(N)

# summ progressed if trtrand==0

# return scalar prog_number_con=r(mean)*r(N)

# return scalar con_number=r(N)

# summ cens if trtrand==0 & xo==1

# return scalar cens_number_xo=r(mean)*r(N)

# ***estimate HR and survival using FPM***

# stset timeOS2, failure(died) id(id)

# capture stpm2 trtrand, scale(h) df(4) lininit iterate(200)

# if e(converged)==. | e(converged)==0 {

# capture stpm2 trtrand, scale(h) df(3) lininit iterate(200)

# if e(converged)==. | e(converged)==0 {

# capture stpm2 trtrand, scale(h) df(2) lininit iterate(200)

# if e(converged)==. | e(converged)==0 {

# capture stpm2 trtrand, scale(h) df(1) lininit iterate(200)

# }

# }

# }

# return scalar itt_fpm_hr = exp(_b[trtrand])

# return scalar itt_fpm_hr_SE = exp(_b[trtrand])*_se[trtrand]

# return scalar itt_fpm_hr_LB = exp((_b[trtrand])-(1.96*_se[trtrand]))

# return scalar itt_fpm_hr_UB = exp((_b[trtrand])+(1.96*_se[trtrand]))

# return scalar zittnl = _b[trtrand] / _se[trtrand]

# gen conv1 = e(converged)

# summ conv1

# return scalar itt_fpm_hr_conv = r(mean)

# capture stpm2 if trtrand==1, scale(h) df(4) lininit iterate(200)

# if e(converged)==. | e(converged)==0 {

# capture stpm2 if trtrand==1, scale(h) df(3) lininit iterate(200)

# if e(converged)==. | e(converged)==0 {

# capture stpm2 if trtrand==1, scale(h) df(2) lininit iterate(200)

# if e(converged)==. | e(converged)==0 {

# capture stpm2 if trtrand==1, scale(h) df(1) lininit iterate(200)

# }

# }

# }

# gen conv2 = e(converged)

# summ conv2

# return scalar exp_fpm_conv = r(mean)

# capture stpm2 if trtrand==0, scale(h) df(4) lininit iterate(200)

# if e(converged)==. | e(converged)==0 {

# capture stpm2 if trtrand==0, scale(h) df(3) lininit iterate(200)

# if e(converged)==. | e(converged)==0 {

# capture stpm2 if trtrand==0, scale(h) df(2) lininit iterate(200)

# if e(converged)==. | e(converged)==0 {

# capture stpm2 if trtrand==0, scale(h) df(1) lininit iterate(200)

# }

# }

# }

# gen conv3 = e(converged)

# capture predict meansurv, rmst tmax(548) ci

# if _rc==498 {

# capture predict meansurv, rmst tmax(548)

# summ meansurv

# return scalar itt_auc_con=r(mean)

# }

# capture predict meansurv2, rmst tmax(548) ci

# if _rc!=498 {

# summ meansurv2

# return scalar itt_auc_con=r(mean)

# summ meansurv2_lci

# return scalar itt_auc_con_LB=r(mean)

# summ meansurv2_uci

# return scalar itt_auc_con_UB=r(mean)

# }

# summ conv3

# return scalar itt_auc_con_conv = r(mean)

# restore

# end

program define tswnew, rclass

version 11

*** 1. TSE WITH WEIBULL AND RECENSORING***

preserve

***check that any switchers died***

collapse (max) trtrand bprog b0 timeOS2 xoOSgainobs xotime died cens timePFSobs admin progressed xo, by(id)

gen check = 1 if died==1 & xo==1

replace check = 0 if check==.

sum check

restore

if r(mean)!=0 {

***Stage 1. Estimate treatment effect in switchers***

gen trtnew=0

replace trtnew=1 if trtrand==0 & xoti==1

replace trtnew=1 if trtrand==1

preserve

drop if trtrand==1

drop if timeOS3<timePFSobs

by id: replace obsno = _n

by id: egen minrisk=min(timeOS3)

by id: replace timeOS3=timeOS3-minrisk

by id: replace xotime=xotime-minrisk

by id: replace timeOS2=timeOS2-minrisk

stset timeOS2, failure(died) id(id)

capture streg trtnew bprog timePFSobs b0 cea1, dist(weibull) time iterate(200)

return scalar weib2m_weib_af = exp(_b[trtnew])

return scalar weib2m_weib_af_SE = exp(_b[trtnew])*_se[trtnew]

return scalar weib2m_weib_af_LB = exp((_b[trtnew])-(1.96*_se[trtnew]))

return scalar weib2m_weib_af_UB = exp((_b[trtnew])+(1.96*_se[trtnew]))

gen conv1 = e(converged)

summ conv1

return scalar weib2m_weib_af_conv = r(mean)

restore

***Stage 2. Shrink survival times in switchers, and re-censor***

sort id

preserve

collapse (max) trtrand bprog b0 timeOS2 xoOSgainobs xotime died cens timePFSobs admin, by(id)

by id: replace timePFSobs = 0 if timePFSobs==.

by id: replace xotime=0 if xotime==.

by id: replace xoOSgainobs=0 if xoOSgainobs==.

gen cfact = timeOS2 if trtrand==1

gen dcfact = died if trtrand==1

replace cfact = (xotime + ((timeOS2-xotime)/return(weib2m_weib_af))) if (trtrand==0 & xotime>0)

replace cfact = timeOS2 if (trtrand==0 & xotime==0)

gen OSadminc = admin/return(weib2m_weib_af) if trtrand==0

replace dcfact = died if trtrand==0

replace dcfact=0 if (OSadminc<=cfact & trtrand==0)

replace cfact = OSadminc if (OSadminc<=cfact & trtrand==0)

***do survival analysis on re-estimated survival times***

stset cfact, failure(dcfact) id(id)

***FPM Analysis for adjusted HR and then RMST***

capture stpm2 trtrand, scale(h) df(4) lininit iterate(200)

if e(converged)==. | e(converged)==0 | _rc!=0 {

capture stpm2 trtrand, scale(h) df(3) lininit iterate(200)

if e(converged)==. | e(converged)==0 | _rc!=0 {

capture stpm2 trtrand, scale(h) df(2) lininit iterate(200)

if e(converged)==. | e(converged)==0 | _rc!=0 {

capture stpm2 trtrand, scale(h) df(1) lininit iterate(200)

}

}

}

if _rc==0 {

return scalar weib2m_adj_fpm_hr = exp(_b[trtrand])

return scalar weib2m_adj_fpm_hr_SE = exp(_b[trtrand])*_se[trtrand]

return scalar weib2m_adj_fpm_hr_LB = exp((_b[trtrand])-(1.96*_se[trtrand]))

return scalar weib2m_adj_fpm_hr_UB = exp((_b[trtrand])+(1.96*_se[trtrand]))

gen conv1 = e(converged)

summ conv1

return scalar weib2m_adj_fpm_conv = r(mean)

}

capture stpm2 if trtrand==0, scale(h) df(4) lininit iterate(200)

if e(converged)==. | e(converged)==0 | _rc!=0 {

capture stpm2 if trtrand==0, scale(h) df(3) lininit iterate(200)

if e(converged)==. | e(converged)==0 | _rc!=0 {

capture stpm2 if trtrand==0, scale(h) df(2) lininit iterate(200)

if e(converged)==. | e(converged)==0 | _rc!=0 {

capture stpm2 if trtrand==0, scale(h) df(1) lininit iterate(200)

}

}

}

if _rc==0 {

gen conv2 = e(converged)

predict meansurv, rmst tmax(548)

summ meansurv

return scalar weib2m_adj_auc_con=r(mean)

summ conv2

return scalar weib2m_adj_fpm_con_conv = r(mean)

}

restore

***Note: in selected scenarios bootstrap for confidence intervals***

*** 2. TSE WITH WEIBULL AND WITH IPCW INSTEAD OF RECENSORING ***

sort id

preserve

***Stage 2. Shrink survival times in switchers, without re-censoring***

by id: replace timePFSobs = 0 if timePFSobs==.

by id: replace xotime=0 if xotime==.

by id: replace xoOSgainobs=0 if xoOSgainobs==.

by id: egen timeOS4 = max(timeOS2)

by id: gen cfact = timeOS4 if trtrand==1

by id: gen dcfact = died if trtrand==1

by id: replace cfact = (xotime + ((timeOS4-xotime)/return(weib2m_weib_af))) if (trtrand==0 & xotime>0)

by id: replace cfact = timeOS4 if (trtrand==0 & xotime==0)

by id: replace dcfact = died if trtrand==0

gen AF=return(weib2m_weib_af)

if AF<1.0 {

expand 2 if cfact>timeOS4, gen(dup1)

sort id dup1 obsno

by id: gen timeOS5 = 0

by id: replace timeOS5 = timeOS5[_n-1] + 21 if _n!=1

by id: drop if timeOS5>cfact & dup1==1

by id: replace timeOS3 = timeOS5 if dup1==1

by id: replace obsno = _n

by id: replace obscea = obscea[_n-1] if dup1==1

by id: egen death2 = max(died)

by id: replace finalobs=0

by id: replace finalobs = 1 if _n==_N

by id: replace died=0

by id: replace died=1 if finalobs==1 & death2==1

by id: replace died=. if finalobs==1 & death2==0

}

by id: replace progressed =0 if timeOS3<timePFSobs

***Apply IPCW to counterfactual dataset***

by id: egen death = max(died)

by id: drop if timeOS3 >(cfact)

by id: replace finalobs = 0

by id: replace finalobs = 1 if _n==_N

by id: replace died=1 if finalobs==1 & death==1

by id: replace died=. if finalobs==1 & death==0

gen infcensOS=0

replace infcensOS=1 if died==. & trtrand==0 & finalobs==1

***Use splines for time, need to decipher how many knots to use...***

summ timeOS3 if infcens==1

return scalar mink = r(min)

return scalar maxk = r(max)

_pctile timeOS3 if infcens==1, percentile(33,66)

if r(r1)== return(mink) {

rcsgen timeOS3, df(2) if2(infcens==1) gen(spline)

}

_pctile timeOS3 if infcens==1, percentile(33,66)

if r(r2)==return(maxk) {

rcsgen timeOS3, df(2) if2(infcens==1) gen(spline)

}

_pctile timeOS3 if infcens==1, percentile(33,66)

if r(r1)!=return(mink) & r(r2)!= return(maxk) {

rcsgen timeOS3, df(3) if2(infcens==1) gen(spline)

}

***Denominator of weight***

sort id obsno

capture logistic infcensOS bprog b0 progressed timePFSobs obscea spline* if trtrand==0, iterate(200)

gen conv1 = e(converged)

summ conv1

return scalar tsw_ipcw1_conv = r(mean)

gen omit1 = e(k_autoCns)

summ omit1

return scalar tsw_ipcw1_omit = r(mean)

gen cds1 = e(N_cds)

summ cds1

return scalar tsw_ipcw1_cds = r(mean)

gen cdf1 = e(N_cdf)

summ cdf1

return scalar tsw_ipcw1_cdf = r(mean)

predict ptrtrec if e(sample), pr

***Estimate probability of remaining uncensored***

replace ptrtrec=ptrtrec*infcensOS+(1-ptrtrec)*(1-infcensOS)

replace ptrtrec=1 if ptrtrec==.

***Estimate each individual’s probability of their complete censoring history up to each day***

sort id obsno

by id: replace ptrtrec=ptrtrec*ptrtrec[_n-1] if _n!=1

rename ptrtrec censdenom

***Use unstabilised weight (use stabilised in selected scenarios)***

gen weightxo=1/censdenom

replace weightxo=1 if trtrand==1

***Use weights to estimate adjusteed HR (from pooled logistic regression)***

rcsgen timeOS3, df(4) gen(splineOS)

capture logistic died trtrand bprog b0 splineOS*[pw=weightxo], cluster(id) iterate(200)

return scalar tsw_ipcw_cox_hr = exp(_b[trtrand])

return scalar tsw_ipcw_cox_hr_SE = exp(_b[trtrand])*_se[trtrand]

return scalar tsw_ipcw_cox_hr_LB = exp((_b[trtrand])-(1.96*_se[trtrand]))

return scalar tsw_ipcw_cox_hr_UB = exp((_b[trtrand])+(1.96*_se[trtrand]))

gen conv3 = e(converged)

summ conv3

return scalar tsw_ipcw3_conv = r(mean)

gen omit3 = e(k_autoCns)

summ omit3

return scalar tsw_ipcw3_omit = r(mean)

gen cds3 = e(N_cds)

summ cds3

return scalar tsw_ipcw3_cds = r(mean)

gen cdf3 = e(N_cdf)

summ cdf3

return scalar tsw_ipcw3_cdf = r(mean)

***weights diagnostics***

summ weightxo if died!=. & trtrand==0

return scalar tsw_ipcw_weight_min = r(min)

return scalar tsw_ipcw_weight_max = r(max)

return scalar tsw_ipcw_weight_mean = r(mean)

return scalar tsw_ipcw_weight_sd = r(sd)

return scalar tsw_ipcw_weight_cv = r(sd)/r(mean)

***weighted RMST***

stset timeOS2 died if infcens==0 [iw=weightxo], time0(timeOS3)

capture stpm2 if trtrand==0, scale(h) df(4) lininit iterate(200)

if e(converged)==. | e(converged)==0 | _rc!=0 {

capture stpm2 if trtrand==0, scale(h) df(3) lininit iterate(200)

if e(converged)==. | e(converged)==0 | _rc!=0 {

capture stpm2 if trtrand==0, scale(h) df(2) lininit iterate(200)

if e(converged)==. | e(converged)==0 | _rc!=0 {

capture stpm2 if trtrand==0, scale(h) df(1) lininit iterate(200)

}

}

}

if _rc==0 {

gen conv2 = e(converged)

predict meansurv, rmst tmax(548)

summ meansurv

return scalar tsw_ipcw_adj_auc_con=r(mean)

summ conv2

return scalar tsw_ipcw_adj_auc_conv = r(mean)

}

restore

***Note: in selected scenarios bootstrap for confidence intervals***

*** 3. TSE WITH WEIBULL AND WITHOUT RECENSORING ***

sort id

preserve

***Stage 2. Shrink survival times in switchers, without re-censoring***

collapse (max) trtrand bprog b0 timeOS2 xoOSgainobs xotime died cens timePFSobs admin, by(id)

by id: replace timePFSobs = 0 if timePFSobs==.

by id: replace xotime=0 if xotime==.

by id: replace xoOSgainobs=0 if xoOSgainobs==.

gen cfact = timeOS2 if trtrand==1

gen dcfact = died if trtrand==1

replace cfact = (xotime + ((timeOS2-xotime)/return(weib2m_weib_af))) if (trtrand==0 & xotime>0)

replace cfact = timeOS2 if (trtrand==0 & xotime==0)

replace dcfact = died if trtrand==0

***do survival analysis on re-estimated survival times***

stset cfact, failure(dcfact) id(id)

***FPM Analysis for adjusted HR and then RMST***

capture stpm2 trtrand, scale(h) df(4) lininit iterate(200)

if e(converged)==. | e(converged)==0 | _rc!=0 {

capture stpm2 trtrand, scale(h) df(3) lininit iterate(200)

if e(converged)==. | e(converged)==0 | _rc!=0 {

capture stpm2 trtrand, scale(h) df(2) lininit iterate(200)

if e(converged)==. | e(converged)==0 | _rc!=0 {

capture stpm2 trtrand, scale(h) df(1) lininit iterate(200)

}

}

}

if _rc==0 {

return scalar weib2m_adj_fpm_hrnr = exp(_b[trtrand])

return scalar weib2m_adj_fpm_hrnr_SE = exp(_b[trtrand])*_se[trtrand]

return scalar weib2m_adj_fpm_hrnr_LB = exp((_b[trtrand])-(1.96*_se[trtrand]))

return scalar weib2m_adj_fpm_hrnr_UB = exp((_b[trtrand])+(1.96*_se[trtrand]))

gen conv1 = e(converged)

summ conv1

return scalar weib2m_adj_fpm_convnr = r(mean)

}

capture stpm2 if trtrand==0, scale(h) df(4) lininit iterate(200)

if e(converged)==. | e(converged)==0 | _rc!=0 {

capture stpm2 if trtrand==0, scale(h) df(3) lininit iterate(200)

if e(converged)==. | e(converged)==0 | _rc!=0 {

capture stpm2 if trtrand==0, scale(h) df(2) lininit iterate(200)

if e(converged)==. | e(converged)==0 | _rc!=0 {

capture stpm2 if trtrand==0, scale(h) df(1) lininit iterate(200)

}

}

}

if _rc==0 {

gen conv2 = e(converged)

predict meansurv, rmst tmax(548)

summ meansurv

return scalar weib2m_adj_aucnr_con=r(mean)

summ conv2

return scalar weib2m_adj_fpm_con_convnr = r(mean)

}

restore

***Note: in selected scenarios bootstrap for confidence intervals***

}

end

# Appendix C: Treatment switching probabilities

Table C1 presents the probability of switching for different patient groups at different time-points in Scenarios 18, 20, 26 and 28. Higher group numbers represent higher values for that group (that is, ‘time to progression group’ 0 are the control group patients that had time-to-progression times in the lowest 33.3% of the control group). Note however that these groups only refer to patients who became ‘at-risk’ of switching – that is, those control group patients that survived for longer than 21 days. Hence the lowest 33% represent the lowest third of the at-risk group, not the control group as a whole. Switching could happen at the three consultations immediately following disease progression, with the probability of switching declining in each consultation.

Table C1: Probability of treatment switch by prognostic groups and consultation – Good prognosis more likely to switch. Scenarios 18, 20, 26 and 28

| Consultation 1 (post progression) | | Biomarker group at progression | | |
| --- | --- | --- | --- | --- |
|  |  | 0 | 1 | 2 |
| Time to progression group | 0 | 0.06 | 0.11 | 0.18 |
|  | 1 | 0.17 | 0.29 | 0.42 |
|  | 2 | 0.35 | 0.52 | 0.65 |
| Consultation 2 (post progression) | | Biomarker group at progression | | |
|  |  | 0 | 1 | 2 |
| Time to progression group | 0 | 0.05 | 0.09 | 0.15 |
|  | 1 | 0.14 | 0.25 | 0.36 |
|  | 2 | 0.30 | 0.46 | 0.60 |
| Consultation 3 (post progression) | | Biomarker group at progression | | |
|  |  | 0 | 1 | 2 |
| Time to progression group | 0 | 0.03 | 0.06 | 0.10 |
|  | 1 | 0.09 | 0.17 | 0.26 |
|  | 2 | 0.21 | 0.35 | 0.49 |

In Scenario 20 the mean switching proportion in the control group across the 1,000 simulations was 39.5%, which was equivalent to 57.2% of control group patients who became at-risk of switching – i.e. those that experienced disease progression.

Table C2 presents the probability of switching for different patient groups at different time-points in Scenarios 22, 24, 30 and 32. In these scenarios, poor prognosis patients were more likely to switch. In Scenario 9 the mean switching proportion in the control group across the 1,000 simulations was 38.5%, which was equivalent to 55.8% of control group patients who became at-risk of switching – i.e. those that experienced disease progression.

Table C2: Probability of treatment switch by prognostic groups and consultation – Poor prognosis more likely to switch. Scenarios 22, 24, 30 and 32

| Consultation 1 (post progression) | | Biomarker group at progression | | |
| --- | --- | --- | --- | --- |
|  |  | 0 | 1 | 2 |
| Time to progression group | 0 | 0.80 | 0.67 | 0.55 |
|  | 1 | 0.53 | 0.36 | 0.25 |
|  | 2 | 0.20 | 0.11 | 0.07 |
| Consultation 2 (post progression) | | Biomarker group at progression | | |
|  |  | 0 | 1 | 2 |
| Time to progression group | 0 | 0.76 | 0.62 | 0.49 |
|  | 1 | 0.47 | 0.31 | 0.21 |
|  | 2 | 0.17 | 0.09 | 0.06 |
| Consultation 3 (post progression) | | Biomarker group at progression | | |
|  |  | 0 | 1 | 2 |
| Time to progression group | 0 | 0.67 | 0.50 | 0.38 |
|  | 1 | 0.36 | 0.22 | 0.14 |
|  | 2 | 0.11 | 0.06 | 0.04 |

All probabilities in Table A1 and Table A2 were decreased when investigating lower switching scenarios (i.e. in Scenarios 17, 19, 21, 23, 25, 27, 29, 31. Probabilities were adjusted in Scenarios 1-16 and 33--48 in order to maintain similar average switch proportions when survival distributions were either simple (Scenarios 1-16) or more complex (Scenarios 33-48).

# Appendix D: Scenario parameter values

In Table D1, values for each variable in Scenario 1 are quoted, as are alternative values for the other scenarios.

Table D1: Simulated scenarios – Parameter values and alternatives tested

| Variable | Value (Scenario 1) | Alternative Values |
| --- | --- | --- |
| Sample size | 500 (2:1 randomisation) | - |
| Number of prognosis groups (prog) | 2 | - |
| Probability of good prognosis | 0.5 | - |
| Probability of poor prognosis | 0.5 | - |
| Maximum follow-up time | 1.5 years | - |
| Impact of bad prognosis on survival | Log hazard ratio = 0.3 | - |
| Survival time distribution | Weibull parameters:  Scale parameter 0.00001  Shape parameter 1.8 | Weibull parameters in scenarios with high severity:  Scale parameter 0.00002  Shape parameter 1.8  Scenarios with moderate complexity of survivor function  Weibull parameters (low severity):  Mix 1: Scale parameter 0.00001  Shape parameter 2.0  Mix 2: Scale parameter 0.00001  Shape parameter 0.8  p = 0.5 (mix parameter)  Weibull parameters (high severity):  Mix 1: Scale parameter 0.00004  Shape parameter 2.0  Mix 2: Scale parameter 0.00004  Shape parameter 1.5  p = 0.5 (mix parameter)  Scenarios with high complexity of survivor function  Weibull parameters (low severity):  Mix 1: Scale parameter 0.00001  Shape parameter 2.0  Mix 2: Scale parameter 0.00001  Shape parameter 0.8  p = 0.5 (mix parameter)  Weibull parameters (high severity):  Mix 1: Scale parameter 0.00004  Shape parameter 2.0  Mix 2: Scale parameter 0.00004  Shape parameter 1.5  p = 0.5 (mix parameter) |
| Progression free survival | Overall survival time multiplied by a value from a beta distribution with shape parameters (5,10) – this implies the assumption that time to progression is 33% of OS. This is not an important assumption – time to progression is only included because we model a situation where switching cannot occur before disease progression | - |
| Baseline treatment effect (note this is not the true total treatment effect as this does not take into account the effect of the treatment that occurs through the time-dependent confounder, biomarker level, or the time-dependent part of the treatment effect, η ) | Baseline log hazard ratio -0.22 for a small treatment effect | Alter log hazard ratio to -0.60 for a high treatment effect  Scenarios with moderate treatment effect time-dependency (moderately complex survivor function)  Baseline log hazard ratio in scenarios that include an additional time-dependent effect = -1.30  Alter log hazard ratio to -1.10 to maintain treatment effect with more severe disease  Alter log hazard ratio to -0.80 to represent a smaller treatment effect  Alter log hazard ratio to -0.65 to maintain smaller treatment effect with more severe disease  Scenarios with strong treatment effect time-dependency (highly complex survivor function)  Alter log hazard ratio to -2.20 for a high treatment effect  Alter log hazard ratio to -1.8 to maintain treatment effect with more severe disease  Alter log hazard ratio to -1.85 to represent a smaller treatment effect with moderate disease  Alter log hazard ratio to -1.35 to maintain smaller treatment effect with more severe disease |
| Biomarker intercept | Calculated using a normal distribution with mean of 20 and standard deviation of 1. Increased by 2.5 in patients who are in the poor prognosis group. | - |
| Biomarker value progression over time | As demonstrated by Equation (3). $\beta_{2}=-0.02$ to represent that the biomarker value increases more slowly in the experimental group, and $\beta_{1}=0.04$ to indicate that the biomarker value increases over time | - |
| Impact of biomarker value on overall survival | As demonstrated by Equation (6).  $\alpha=0$in scenarios with a constant treatment effect (i.e. those with a simple survivor function) | Increased biomarker value increases the risk of death. The strength of this relationship depends on the variable *α*, which equals 0.01 in scenarios with a moderate or complex survivor function. |
| Impact of biomarker value on treatment effect | $\alpha=0$in scenarios with a constant (zero time-dependency) treatment effect, hence the biomarker does not have an impact on the treatment effect | In scenarios with a moderately or highly complex survivor function treatment reduces the progression of the biomarker value and increased biomarker values increase the risk of death, hence the treatment has an additional effect through the biomarker. The strength of this relationship depends on the variable *α*, which equals 0.01 in scenarios with a moderate or complex survivor function.  All scenarios with a moderate or complex survivor function include a time-dependent treatment effect in the experimental group. However, in selected scenarios the treatment effect received by switchers equals the average treatment effect in the experimental group, satisfying the ‘common treatment effect’ assumption. |
| Time-dependent portion of treatment effect, η | *η* =0 in scenarios with a constant (zero time-dependency) treatment effect | All scenarios with a moderate or complex survivor function include a time-dependent treatment effect in the experimental group. However, in selected scenarios the treatment effect received by switchers equals the average treatment effect in the experimental group, satisfying the ‘common treatment effect’ assumption  *η* =0.0025 to generate a reduction in the treatment effect over time in scenarios with a small treatment effect and a moderately complex survivor function/treatment effect time dependency.  *η* =0.003 to generate a reduction in the treatment effect over time in scenarios with a high treatment effect and a moderately complex survivor function/treatment effect time dependency.  *η* =0.006 to generate a reduction in the treatment effect over time in scenarios with a highly complex survivor function/stronger treatment effect time dependency |
| Assumed frequency of consultations | One every 3 weeks (21 days) | - |
| Probability of switching treatment over time | As shown in Table A1. This results in a switching proportion of approximately 24% in Scenario 1 | Test a high switching scenario where all probabilities are increased – to an extent where approximately 50% of control group patients switch. |
| Prognosis of switching patients | As shown in Table A1. This makes switching more likely in good prognosis patients, via a mechanism that takes into account both time to progression and biomarker value at progression | As shown in Table A2. This makes switching more likely in poor prognosis patients, via a mechanism that takes into account both time to progression and biomarker value at progression |
| Treatment effect in switching patients | Equal to baseline treatment effect multiplied by *ω*. Set *ω* such that treatment effect received by switching patients is 100% of the average effect received by experimental group patients in base scenarios. | - |

# Appendix E: Scenario settings

| Scenario | Switch proportion | Treatment effect | Switcher prognosis | Severity of disease | Complexity of survivor function and time-dependency of treatment effect |
| --- | --- | --- | --- | --- | --- |
| 1 | Low | Low | Good | Low | Simple / Zero |
| 2 | Moderate | Low | Good | Low | Simple / Zero |
| 3 | Low | High | Good | Low | Simple / Zero |
| 4 | Moderate | High | Good | Low | Simple / Zero |
| 5 | Low | Low | Poor | Low | Simple / Zero |
| 6 | Moderate | Low | Poor | Low | Simple / Zero |
| 7 | Low | High | Poor | Low | Simple / Zero |
| 8 | Moderate | High | Poor | Low | Simple / Zero |
| 9 | Low | Low | Good | High | Simple / Zero |
| 10 | Moderate | Low | Good | High | Simple / Zero |
| 11 | Low | High | Good | High | Simple / Zero |
| 12 | Moderate | High | Good | High | Simple / Zero |
| 13 | Low | Low | Poor | High | Simple / Zero |
| 14 | Moderate | Low | Poor | High | Simple / Zero |
| 15 | Low | High | Poor | High | Simple / Zero |
| 16 | Moderate | High | Poor | High | Simple / Zero |
| 17 | Low | Low | Good | Low | Mixture / Moderate |
| 18 | Moderate | Low | Good | Low | Mixture / Moderate |
| 19 | Low | High | Good | Low | Mixture / Moderate |
| 20 | Moderate | High | Good | Low | Mixture / Moderate |
| 21 | Low | Low | Poor | Low | Mixture / Moderate |
| 22 | Moderate | Low | Poor | Low | Mixture / Moderate |
| 23 | Low | High | Poor | Low | Mixture / Moderate |
| 24 | Moderate | High | Poor | Low | Mixture / Moderate |
| 25 | Low | Low | Good | High | Mixture / Moderate |
| 26 | Moderate | Low | Good | High | Mixture / Moderate |
| 27 | Low | High | Good | High | Mixture / Moderate |
| 28 | Moderate | High | Good | High | Mixture / Moderate |
| 29 | Low | Low | Poor | High | Mixture / Moderate |
| 30 | Moderate | Low | Poor | High | Mixture / Moderate |
| 31 | Low | High | Poor | High | Mixture / Moderate |
| 32 | Moderate | High | Poor | High | Mixture / Moderate |
| 33 | Low | Low | Good | Low | Mixture / Strong |
| 34 | Moderate | Low | Good | Low | Mixture / Strong |
| 35 | Low | High | Good | Low | Mixture / Strong |
| 36 | Moderate | High | Good | Low | Mixture / Strong |
| 37 | Low | Low | Poor | Low | Mixture / Strong |
| 38 | Moderate | Low | Poor | Low | Mixture / Strong |
| 39 | Low | High | Poor | Low | Mixture / Strong |
| 40 | Moderate | High | Poor | Low | Mixture / Strong |
| 41 | Low | Low | Good | High | Mixture / Strong |
| 42 | Moderate | Low | Good | High | Mixture / Strong |
| 43 | Low | High | Good | High | Mixture / Strong |
| 44 | Moderate | High | Good | High | Mixture / Strong |
| 45 | Low | Low | Poor | High | Mixture / Strong |
| 46 | Moderate | Low | Poor | High | Mixture / Strong |
| 47 | Low | High | Poor | High | Mixture / Strong |
| 48 | Moderate | High | Poor | High | Mixture / Strong |

# Appendix F: Overview of simulation scenarios

Table F1 presents key details associated with each of the scenarios simulated. The true area under the curve (restricted mean survival time (RMST) at 548 days) unconfounded by treatment switching is presented, along with the average treatment effect in terms of a hazard ratio (HR) and an acceleration factor (AF). These were estimated by generating scenario data for 1,000,000 patients without applying switching – RMST was estimated directly from this data, Cox models were used to estimate the HR, and an RPSFTM under no switching was used to estimate the AF. The HR and AF represent only an approximation of the true treatment effect as the proportional hazards and constant acceleration factor assumptions do not hold. In terms of a hazard ratio, the average treatment effect varied between 0.54 and 0.81. We also indicate the extent to which the treatment effect changed over time.

The proportion of control group patients that switched, averaged across the 1000 simulations that made up each scenario, is also presented. The switching proportion varied between 17% and 57% of all control group patients. Switching proportions are probabilistic and are reliant on other characteristics. Table F1 also presents the switching proportion as a percentage of the control group patients that became ‘at-risk’ of switching. In our simulations control group patients could only switch treatments if they were alive at their first ‘consultation’ at 21 days and if their disease progressed before the end of the simulated follow-up. The switching proportion as a percentage of patients that became at-risk of switching is higher than when it is measured as a percentage of all control group patients – it ranged from 21% to 58%. We estimated the proportion of patients who became at risk of switching in each scenario by collecting data on the number of patients for whom disease progression was observed in each simulation and taking the mean. This is approximate, but appropriately indicative for our purposes.

Table F1 also presents details on the mean proportion of patients that were censored in each scenario – that is, the proportion for whom death was not observed. This varied between 10% and 58%.

Table F1: Overview of simulated scenarios

| Scenario | Switch proportion | Treatment effect | Switcher prognosis | Severity of disease | Complexity of survivor function | Truth (years) | | Average treatment effects | | Treatment effect in switchers (AF) | Mean switcher % of total | Mean switcher % of at risk | Mean censoring proportion (%) |
| --- | --- | --- | --- | --- | --- | --- | --- | --- | --- | --- | --- | --- | --- |
|  |  |  |  |  |  | RMST (Control group) | RMST (Exp group) | HR | AF |  |  |  |  |
| 1 | Low | Low | Good | Low | Simple | 400.80 | 423.33 | 0.80 | 1.13 | 1.13 | 0.24 | 0.24 | 0.47 |
| 2 | Moderate | Low | Good | Low | Simple | 400.80 | 423.33 | 0.80 | 1.13 | 1.13 | 0.54 | 0.56 | 0.47 |
| 3 | Low | High | Good | Low | Simple | 400.80 | 456.60 | 0.55 | 1.40 | 1.40 | 0.24 | 0.24 | 0.56 |
| 4 | Moderate | High | Good | Low | Simple | 400.80 | 456.60 | 0.55 | 1.40 | 1.40 | 0.53 | 0.55 | 0.57 |
| 5 | Low | Low | Poor | Low | Simple | 400.80 | 423.33 | 0.80 | 1.13 | 1.13 | 0.27 | 0.28 | 0.47 |
| 6 | Moderate | Low | Poor | Low | Simple | 400.80 | 423.33 | 0.80 | 1.13 | 1.13 | 0.56 | 0.58 | 0.48 |
| 7 | Low | High | Poor | Low | Simple | 400.80 | 456.60 | 0.55 | 1.40 | 1.40 | 0.27 | 0.28 | 0.56 |
| 8 | Moderate | High | Poor | Low | Simple | 400.80 | 456.60 | 0.55 | 1.40 | 1.40 | 0.56 | 0.58 | 0.58 |
| 9 | Low | Low | Good | High | Simple | 315.41 | 343.95 | 0.80 | 1.13 | 1.13 | 0.24 | 0.25 | 0.23 |
| 10 | Moderate | Low | Good | High | Simple | 315.41 | 343.95 | 0.80 | 1.13 | 1.13 | 0.56 | 0.56 | 0.23 |
| 11 | Low | High | Good | High | Simple | 315.41 | 390.14 | 0.55 | 1.40 | 1.40 | 0.24 | 0.25 | 0.32 |
| 12 | Moderate | High | Good | High | Simple | 315.41 | 390.14 | 0.55 | 1.40 | 1.40 | 0.55 | 0.56 | 0.34 |
| 13 | Low | Low | Poor | High | Simple | 315.41 | 343.95 | 0.80 | 1.13 | 1.13 | 0.26 | 0.26 | 0.22 |
| 14 | Moderate | Low | Poor | High | Simple | 315.41 | 343.95 | 0.80 | 1.13 | 1.13 | 0.57 | 0.57 | 0.23 |
| 15 | Low | High | Poor | High | Simple | 315.41 | 390.14 | 0.55 | 1.40 | 1.40 | 0.26 | 0.26 | 0.31 |
| 16 | Moderate | High | Poor | High | Simple | 315.41 | 390.14 | 0.55 | 1.40 | 1.40 | 0.57 | 0.57 | 0.33 |
| 17 | Low | Low | Good | Low | Moderate | 357.46 | 391.12 | 0.81 | 1.19 | 1.19 | 0.17 | 0.25 | 0.40 |
| 18 | Moderate | Low | Good | Low | Moderate | 357.46 | 391.12 | 0.81 | 1.19 | 1.19 | 0.39 | 0.57 | 0.40 |
| 19 | Low | High | Good | Low | Moderate | 357.46 | 430.07 | 0.57 | 1.53 | 1.53 | 0.17 | 0.25 | 0.48 |
| 20 | Moderate | High | Good | Low | Moderate | 357.46 | 430.07 | 0.57 | 1.53 | 1.53 | 0.40 | 0.57 | 0.50 |
| 21 | Low | Low | Poor | Low | Moderate | 357.46 | 391.12 | 0.81 | 1.19 | 1.19 | 0.17 | 0.24 | 0.39 |
| 22 | Moderate | Low | Poor | Low | Moderate | 357.46 | 391.12 | 0.81 | 1.19 | 1.19 | 0.39 | 0.56 | 0.40 |
| 23 | Low | High | Poor | Low | Moderate | 357.46 | 430.07 | 0.57 | 1.53 | 1.53 | 0.17 | 0.24 | 0.47 |
| 24 | Moderate | High | Poor | Low | Moderate | 357.46 | 430.07 | 0.57 | 1.53 | 1.53 | 0.38 | 0.56 | 0.48 |
| 25 | Low | Low | Good | High | Moderate | 228.38 | 269.20 | 0.78 | 1.30 | 1.30 | 0.24 | 0.25 | 0.18 |
| 26 | Moderate | Low | Good | High | Moderate | 228.38 | 269.20 | 0.78 | 1.30 | 1.30 | 0.55 | 0.57 | 0.18 |
| 27 | Low | High | Good | High | Moderate | 228.38 | 322.24 | 0.56 | 1.85 | 1.85 | 0.24 | 0.25 | 0.25 |
| 28 | Moderate | High | Good | High | Moderate | 228.38 | 322.24 | 0.56 | 1.85 | 1.85 | 0.55 | 0.57 | 0.26 |
| 29 | Low | Low | Poor | High | Moderate | 228.38 | 269.20 | 0.78 | 1.30 | 1.30 | 0.20 | 0.21 | 0.17 |
| 30 | Moderate | Low | Poor | High | Moderate | 228.38 | 269.20 | 0.78 | 1.30 | 1.30 | 0.51 | 0.53 | 0.17 |
| 31 | Low | High | Poor | High | Moderate | 228.38 | 322.24 | 0.56 | 1.85 | 1.85 | 0.20 | 0.21 | 0.24 |
| 32 | Moderate | High | Poor | High | Moderate | 228.38 | 322.24 | 0.56 | 1.85 | 1.85 | 0.51 | 0.53 | 0.24 |
| 33 | Low | Low | Good | Low | High | 357.46 | 405.74 | 0.78 | 1.21 | 1.21 | 0.17 | 0.25 | 0.35 |
| 34 | Moderate | Low | Good | Low | High | 357.46 | 405.74 | 0.78 | 1.21 | 1.21 | 0.39 | 0.57 | 0.36 |
| Scenario | Switch proportion | Treatment effect | Switcher prognosis | Severity of disease | Complexity of survivor function | Truth (years) | | Average treatment effect | | Treatment effect in switchers (AF) | Mean switcher % of total | Mean switcher % of at risk | Mean censoring proportion (%) |
|  |  |  |  |  |  | RMST (Control group) | RMST (Exp group) | HR | AF |  |  |  |  |
| 35 | Low | High | Good | Low | High | 357.46 | 438.20 | 0.56 | 1.52 | 1.52 | 0.17 | 0.25 | 0.44 |
| 36 | Moderate | High | Good | Low | High | 357.46 | 438.20 | 0.56 | 1.52 | 1.41 | 0.40 | 0.57 | 0.46 |
| 37 | Low | Low | Poor | Low | High | 357.46 | 405.74 | 0.78 | 1.21 | 1.21 | 0.17 | 0.24 | 0.35 |
| 38 | Moderate | Low | Poor | Low | High | 357.46 | 405.74 | 0.78 | 1.21 | 1.21 | 0.39 | 0.56 | 0.35 |
| 39 | Low | High | Poor | Low | High | 357.46 | 438.20 | 0.56 | 1.52 | 1.52 | 0.17 | 0.25 | 0.43 |
| 40 | Moderate | High | Poor | Low | High | 357.46 | 438.20 | 0.56 | 1.52 | 1.52 | 0.38 | 0.56 | 0.44 |
| 41 | Low | Low | Good | High | High | 228.38 | 273.39 | 0.81 | 1.23 | 1.23 | 0.24 | 0.25 | 0.11 |
| 42 | Moderate | Low | Good | High | High | 228.38 | 273.39 | 0.81 | 1.23 | 1.23 | 0.55 | 0.58 | 0.11 |
| 43 | Low | High | Good | High | High | 228.38 | 333.63 | 0.54 | 1.85 | 1.85 | 0.24 | 0.25 | 0.18 |
| 44 | Moderate | High | Good | High | High | 228.38 | 333.63 | 0.54 | 1.85 | 1.85 | 0.56 | 0.58 | 0.19 |
| 45 | Low | Low | Poor | High | High | 228.38 | 273.39 | 0.81 | 1.23 | 1.23 | 0.20 | 0.21 | 0.10 |
| 46 | Moderate | Low | Poor | High | High | 228.38 | 273.39 | 0.81 | 1.23 | 1.23 | 0.51 | 0.53 | 0.11 |
| 47 | Low | High | Poor | High | High | 228.38 | 333.63 | 0.54 | 1.85 | 1.85 | 0.20 | 0.21 | 0.18 |
| 48 | Moderate | High | Poor | High | High | 228.38 | 333.63 | 0.54 | 1.85 | 1.85 | 0.51 | 0.53 | 0.18 |

# Appendix G: Percentage bias across all scenarios (note different axis scales)

Figure G1: Percentage bias, Scenarios 1-16


Figure G2: Percentage bias, Scenarios 17-32

Figure G3: Percentage bias, Scenarios 33-48

# Appendix H: Empirical standard error of percentage bias across all scenarios (note different axis scales)

Figure H1: Empirical standard error, Scenarios 1-16


Figure H2: Empirical standard error, Scenarios 17-32


Figure H3: Empirical standard error, Scenarios 33-48

# Appendix I: Root mean squared error of percentage bias across all scenarios (note different axis scales)

Figure I1: Root mean squared error, Scenarios 1-16


Figure I2: Root mean squared error, Scenarios 17-32

Figure I3: Root mean squared error, Scenarios 33-48

# Appendix J: TSEipcw with stabilised and unstabilised weights

Figure J1: Percentage bias, Scenarios 17-20, 25-28


Figure J2: Empirical standard error, Scenarios 17-20, 25-28

Figure J3: Root mean squared error, Scenarios 17-20, 25-28


# Appendix K: Percentage bias, empirical standard error and root mean squared error when simulations with high maximum weights are excluded

Figure K1. Percentage bias across all scenarios, excluding simulations where maximum weight > 1000

Note: ITT: intention to treat; TSE: two-stage estimation; TSEnr: two-stage estimation without re-censoring; TSEipcw: two-stage estimation with inverse probability of censoring weights

Figure K2. Empirical standard error across all scenarios, excluding simulations where maximum weight > 1000


Note: ITT: intention to treat; TSE: two-stage estimation; TSEnr: two-stage estimation without re-censoring; TSEipcw: two-stage estimation with inverse probability of censoring weights. SE: standard error

Figure K3. Root mean squared error across all scenarios, excluding simulations where maximum weight > 1000


Note: ITT: intention to treat; TSE: two-stage estimation; TSEnr: two-stage estimation without re-censoring; TSEipcw: two-stage estimation with inverse probability of censoring weights. RMSE: root mean squared error

Figure K4. Percentage bias across all scenarios, excluding simulations where maximum weight > 100

Note: ITT: intention to treat; TSE: two-stage estimation; TSEnr: two-stage estimation without re-censoring; TSEipcw: two-stage estimation with inverse probability of censoring weights

Figure K5. Empirical standard error across all scenarios, excluding simulations where maximum weight > 100


Note: ITT: intention to treat; TSE: two-stage estimation; TSEnr: two-stage estimation without re-censoring; TSEipcw: two-stage estimation with inverse probability of censoring weights. SE: standard error

Figure K6. Root mean squared error across all scenarios, excluding simulations where maximum weight > 100


Note: ITT: intention to treat; TSE: two-stage estimation; TSEnr: two-stage estimation without re-censoring; TSEipcw: two-stage estimation with inverse probability of censoring weights. RMSE: root mean squared error

Figure K7. Percentage bias across all scenarios, excluding simulations where maximum weight > 20

Note: ITT: intention to treat; TSE: two-stage estimation; TSEnr: two-stage estimation without re-censoring; TSEipcw: two-stage estimation with inverse probability of censoring weights

Figure K8. Empirical standard error across all scenarios, excluding simulations where maximum weight > 20


Note: ITT: intention to treat; TSE: two-stage estimation; TSEnr: two-stage estimation without re-censoring; TSEipcw: two-stage estimation with inverse probability of censoring weights. SE: standard error

Figure K9. Root mean squared error across all scenarios, excluding simulations where maximum weight > 20


Note: ITT: intention to treat; TSE: two-stage estimation; TSEnr: two-stage estimation without re-censoring; TSEipcw: two-stage estimation with inverse probability of censoring weights. RMSE: root mean squared error

# 
